# *Tyro3* Modulates *Mertk*-Associated Retinal Degeneration

**DOI:** 10.1371/journal.pgen.1005723

**Published:** 2015-12-11

**Authors:** Douglas Vollrath, Douglas Yasumura, Gillie Benchorin, Michael T. Matthes, Wei Feng, Natalie M. Nguyen, Cecilia D. Sedano, Melissa A. Calton, Matthew M. LaVail

**Affiliations:** 1 Department of Genetics, Stanford University School of Medicine, Stanford, California, United States of America; 2 Beckman Vision Center, University of California San Francisco, San Francisco, California, United States of America; University of California San Diego, UNITED STATES

## Abstract

Inherited photoreceptor degenerations (IPDs) are the most genetically heterogeneous of Mendelian diseases. Many IPDs exhibit substantial phenotypic variability, but the basis is usually unknown. Mutations in *MERTK* cause recessive IPD phenotypes associated with the RP38 locus. We have identified a murine genetic modifier of *Mertk*-associated photoreceptor degeneration, the C57BL/6 (B6) allele of which acts as a suppressor. Photoreceptors degenerate rapidly in *Mertk*-deficient animals homozygous for the 129P2/Ola (129) modifier allele, whereas animals heterozygous for B6 and 129 modifier alleles exhibit an unusual intermixing of degenerating and preserved retinal regions, with females more severely affected than males. *Mertk*-deficient mice homozygous for the B6 modifier allele display degeneration only in the far periphery, even at 8 months of age, and have improved retinal function compared to animals homozygous for the 129 allele. We genetically mapped the modifier to an approximately 2-megabase critical interval that includes *Tyro3*, a paralog of *Mertk*. *Tyro3* expression in the outer retina varies with modifier genotype in a manner characteristic of a cis-acting expression quantitative trait locus (eQTL), with the B6 allele conferring an approximately three-fold higher expression level. Loss of *Tyro3* function accelerates the pace of photoreceptor degeneration in *Mertk* knockout mice, and TYRO3 protein is more abundant in the retinal pigment epithelium (RPE) adjacent to preserved central retinal regions of *Mertk* knockout mice homozygous for the B6 modifier allele. Endogenous human TYRO3 protein co-localizes with nascent photoreceptor outer segment (POS) phagosomes in a primary RPE cell culture assay, and expression of murine *Tyro3* in cultured cells stimulates phagocytic ingestion of POS. Our findings demonstrate that *Tyro3* gene dosage modulates *Mertk*-associated retinal degeneration, provide strong evidence for a direct role for TYRO3 in RPE phagocytosis, and suggest that an eQTL can modify a recessive IPD.

## Author Summary

Human inherited photoreceptor degenerations (IPDs) are genetically heterogeneous and exhibit significant unexplained phenotypic variability. Mutation of hundreds of known genes can cause these disorders, but variability still occurs among individuals with mutations in the same gene. Possible explanations include environmental factors, mutation-specific effects, and sequence variants of modifier genes that influence the impact of disease-causing mutations. We have identified a modifier locus associated with profound suppression of photoreceptor degeneration in a mouse model of a human IPD. A gene similar to the IPD gene lies within the modifier locus, and the protein encoded by this putative modifier gene seems to substitute for the IPD protein. Our results link modest variation in the quantity of the modifier transcript, and a consequent change in the quantity of the protein, with variation in degeneration severity. Many common human genetic variants cause differences in the transcript levels of individual genes, but a significant fraction of such variants manifest only in subsets of tissues, and ocular tissues have not been surveyed. Our work suggests that variation in the transcript levels of modifier genes may contribute to variability in human IPDs, and that the retina should be assessed for genetic variants that affect gene expression.

## Introduction

Inherited photoreceptor degenerations (IPDs) are the most genetically heterogeneous of Mendelian diseases; more than 200 genes are currently associated with various types (https://sph.uth.edu/retnet/), and new genes continue to be identified. Most IPDs are monogenic, but digenic [1] and oligogenic [2] forms have been described. Monogenic IPDs can exhibit substantial phenotypic variability, in part due to the effects of particular mutations on the function of IPD genes. Genetic modifier loci can also contribute to variability, usually through coding sequence variants [3–5], but in one case of dominant disease, through variation in the expression level of a modifier that in turn modulates the penetrance of an IPD mutation [6]. Given the large number of IPD genes and an abundance of human expression quantitative trait loci (eQTL) [7], one might expect eQTL modification of IPDs to be more common.

Retinitis pigmentosa (RP) is a form of IPD in which photoreceptors degenerate due to mutations in any one of more than 50 genes expressed in the outer retina. *MERTK* is one such gene, expressed adjacent to photoreceptors in the retinal pigment epithelium (RPE) and corresponding to disease phenotypes associated with the RP38 locus. *MERTK* mutations account for ~1% of autosomal recessive RP in most populations, but a *MERTK* founder mutation causes ~30% of RP in the Faroe Islands [8]. *MERTK* was originally associated with retinal disease beginning in the first decade of life [9], and most subsequent studies describe similar ages at onset [10]. In one consanguineous family with *MERTK*-associated RP, the disease progressed more slowly [11], suggesting that genetic modifiers of *MERTK*-associated retinal degeneration exist in the human population.

Mice have long served as useful models for human IPDs. Genetic modification of murine photoreceptor phenotypes has been ascribed to both strain background [12,13] and to coding sequence variants of specific genes [14–16]. Photoreceptor degeneration in mice with loss of *Mertk* function is apparent at a young age [17], consistent with the onset of disease in most humans. RPE cells of these mice have a profound defect in the phagocytosis of photoreceptor outer segment (POS) tips, resulting in a vacuolated, degenerative POS layer and progressive thinning of the outer nuclear layer as photoreceptors die. These features mirror those seen in the Royal College of Surgeons (RCS) rat strain, which harbors a naturally occurring loss-of-function allele of *Mertk* [18], emphasizing the essential role of *Mertk* in mammalian outer retinal homeostasis.

MERTK, along with TYRO3 and AXL, constitute the TAM family of receptor tyrosine kinases. Identification of *Mertk* as the gene mutated in RCS rats was the first connection of the TAM family to the process of phagocytosis. It is now appreciated that TAM receptors function in a diverse array of phagocytic processes including immune system clearance of apoptotic cells [19], Sertoli cell uptake of spermatid residual bodies during spermatogenesis [20], macrophage engulfment of pyrenocytes during erythropoiesis [21], remodeling of the breast epithelium following lactation [22], and astrocytic pruning of neural synapses [23], among others [24].

In contrast to the obvious critical role of MERTK in RPE phagocytosis, there is a lack of consensus regarding the importance of TYRO3 in the retina. Absence of retinal degeneration in *Tyro3* knockout mice led one group to conclude that the gene does not contribute to retinal homeostasis [25], while the presence of TYRO3 protein in the murine RPE led others to posit that it functions there in an unspecified manner [26], but that MERTK has a predominant role in RPE phagocytosis [27]. We address this conundrum by implicating a *Tyro3* eQTL in the nearly complete suppression of the murine IPD associated with loss of *Mertk* function. We demonstrate that *Tyro3* gene dosage modulates the severity of *Mertk*-associated photoreceptor degeneration, and provide evidence that TYRO3 can promote RPE phagocytosis of photoreceptor outer segments in the absence of MERTK. Our results have implications for tissues that co-express MERTK and TYRO3, and highlight the potential for eQTL to impact the phenotypic variability of human IPDs, including phenotypes associated with RP38.

## Results

### Suppression of *Mertk*-associated photoreceptor degeneration by a locus linked to *Mertk*

**Fig 1:**
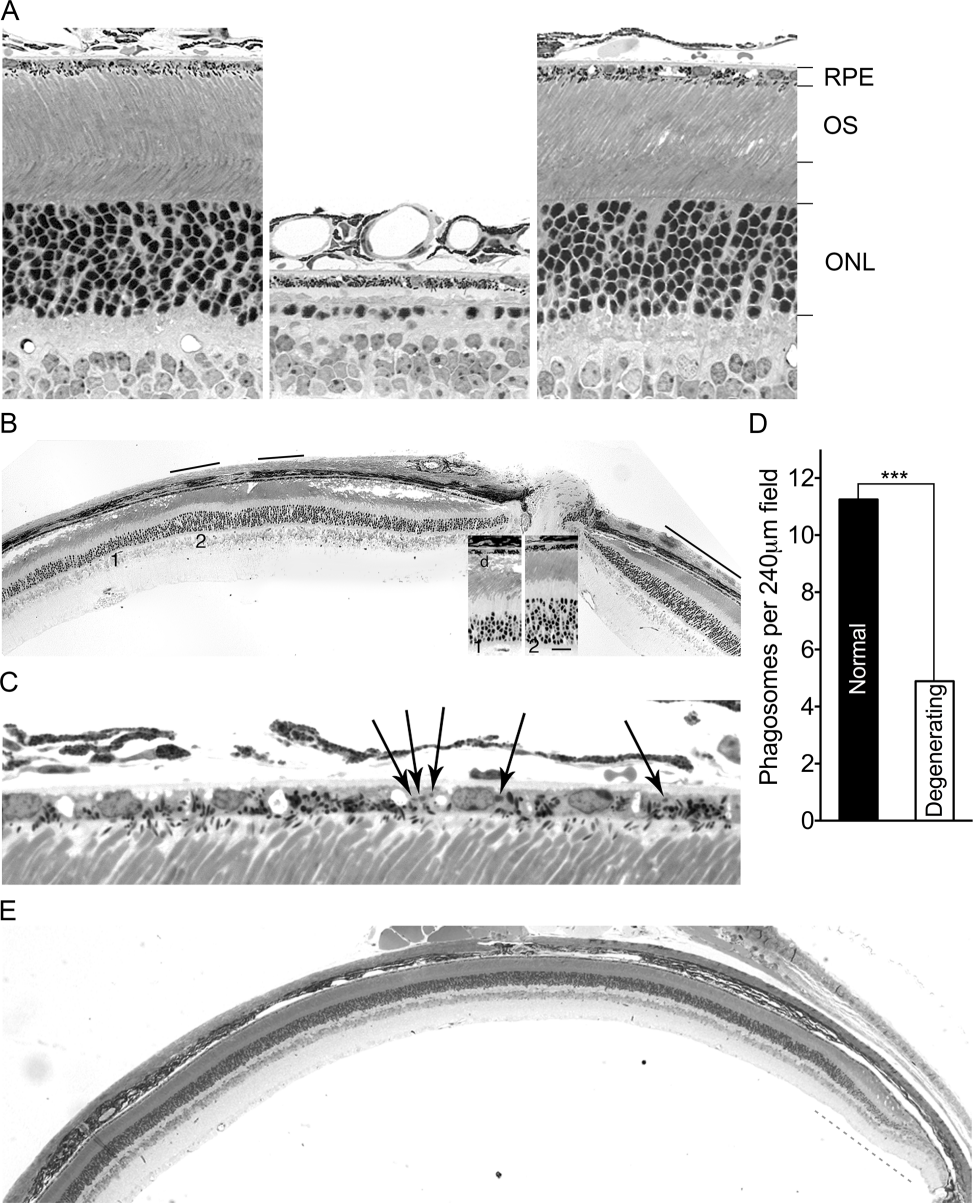
Genetic suppression of photoreceptor degeneration and enhanced RPE phagocytosis in C57BL/6 (B6) incipient congenic *Mertk* ^*-/-*^ mice. (A) Retinal images taken at postnatal day (P) 60 of a wild-type B6 mouse (left), a mouse from the initial *Mertk* knockout line [17] (middle), and a *Mertk* ^*-/-*^ animal with a B6 modifier allele (right) demonstrate striking suppression of photoreceptor degeneration by the modifier. The retinal pigment epithelium (RPE), photoreceptor outer segment (OS) layer, and outer nuclear layer (ONL) are labeled. (B) A low magnification image illustrates intermixing of normal appearing (black bars) and degenerating retinal regions in a P60 *Mertk* ^*-/-*^ mouse heterozygous for a B6 modifier allele linked to *Mertk*. Insets show enlargements of regions of degenerating (1) and normal (2) areas. The ONL of the degenerating region is only 40–50% as thick as that in the normal region. The OS in the normal region are indistinguishable from those of wild-type mice (Fig 1A), whereas in the degenerating region the OS zone is vacuolated and contains disorganized membranous debris (d) characteristic of *Mertk* ^*-/-*^ mice [17]. (C) A higher magnification image from a different animal at P60 shows phagosomes (arrows) in the RPE of a normal-appearing region. (D) Phagosome counts made across full sections at the vertical meridian from five eyes of *Mertk* ^*-/-*^ mice (P104 –P108) heterozygous for the modifier demonstrate increased mean numbers in normal-appearing regions. *** *P* = 0.0004 by an unpaired two-tailed *t*-test. (E) A low magnification image of a retinal section from a *Mertk* ^*-/-*^ mouse homozygous for a B6 modifier allele at P255 demonstrates normal structure across a large extent of the retina, with photoreceptor degeneration in the far periphery (gray dashes) as evidenced by a thinner ONL and a thicker OS layer with vacuoles and debris.

**Fig 2:**
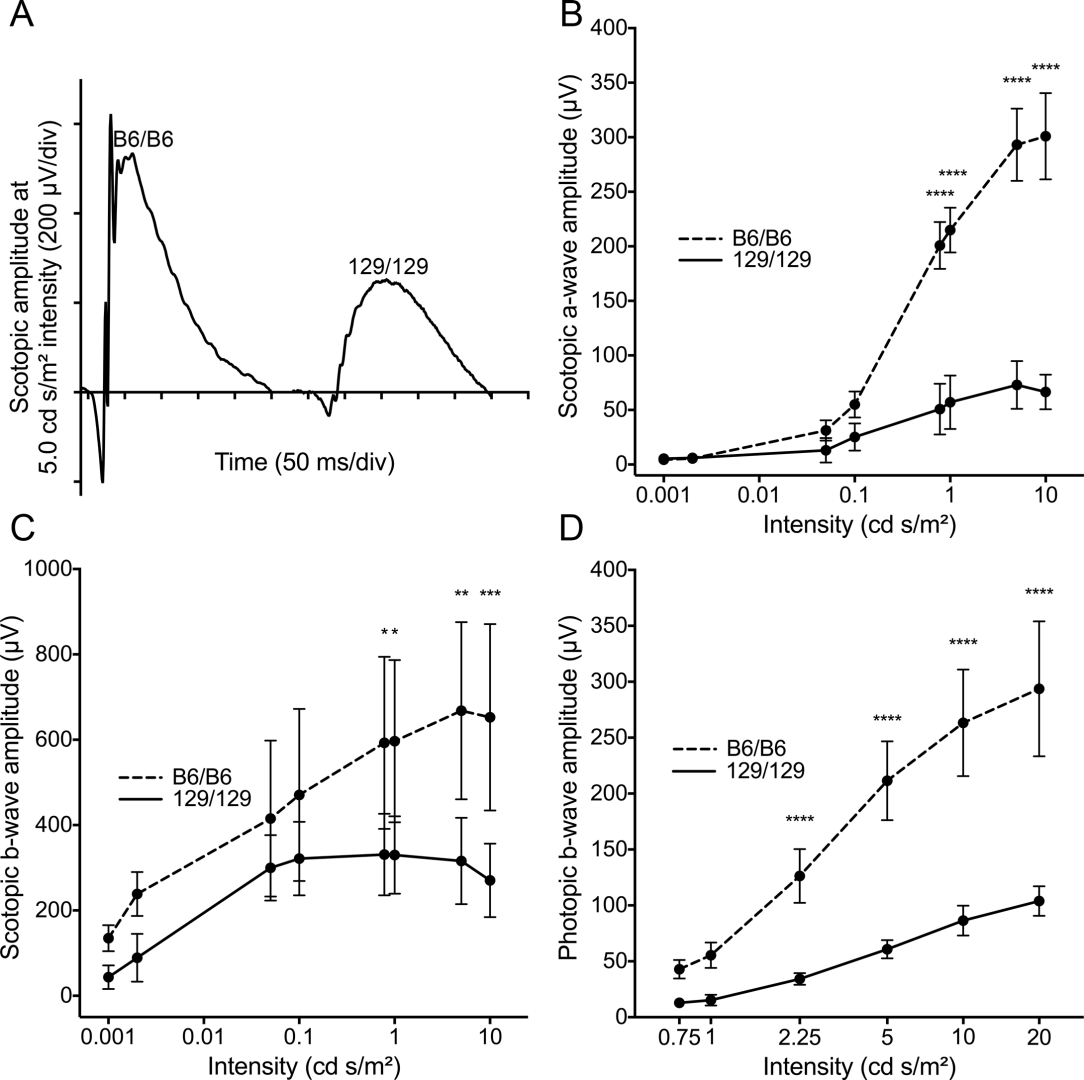
Preservation of photoreceptor function in *Mertk* ^*-/-*^ mice homozygous for a B6 modifier allele. (A) Representative electroretinographic (ERG) waveforms at one light intensity for scotopic (dark-adapted) incipient congenic mice homozygous for either B6 or 129 modifier alleles. Comparison of mean amplitudes ± SD of (B) scotopic a-wave, (C) scotopic b-wave and (D) photopic b-wave at various light intensities for the two lines (n = 5 males at 10–11 weeks for each) demonstrates increased retinal responses in B6 homozygotes. **** *P* ≤ 0.0001, *** *P* ≤ 0.001, ** *P* ≤ 0.01 by a two-way ANOVA with Bonferroni’s correction for multiple comparisons.

After backcrossing the *Mertk* knockout allele [28] from the line we initially characterized [17] onto C57BL/6 (B6) for six more generations, we found a surprising phenotype in a minority of animals–retinal areas that appeared histologically normal at postnatal day (P) 60, an age at which almost all photoreceptors were lost in the original line (Fig 1A). Normal-appearing areas were intermixed with degenerating regions (Fig 1B), and persisted in animals as old as one year (S1 Fig). Phagosomes were evident in the RPE adjacent to normal regions (Fig 1C), and were significantly more numerous in those areas as compared to degenerating regions (Fig 1D). The presence of a suppressor phenotype after numerous backcross generations suggested a modifier locus genetically linked to *Mertk*. We used simple tandem repeat (STR) markers to assess a region of chromosome 2 surrounding the *Mertk* knockout allele in backcrossed mice. As expected from the estimated total number of backcross generations [29], degeneration-susceptible animals harbored an approximately 40 centimorgan segment around *Mertk* that was homozygous for 129P2/Ola (129) alleles (S2A Fig), the strain of origin for the embryonic stem cell line used to generate the *Mertk* knockout allele [28]. Significantly, *Mertk* ^*-/-*^ animals with the suppressor phenotype were heterozygous for 129 and B6 alleles in a region immediately centromere proximal to *Mertk*, indicating the presence of a recombinant chromosome (S2B Fig). The eyes of *Mertk* ^*-/-*^ mice homozygous for this recombinant chromosome were remarkable; most of the retina was histologically normal with degeneration evident only in the periphery, even in animals 8 months of age (Fig 1E). Consistent with these histological findings, electroretinographic (ERG) recordings demonstrated preservation of both rod (scotopic) and cone (photopic) function in animals homozygous for the recombinant chromosome, compared to mice homozygous for 129 alleles in the chromosomal region (Fig 2). These results indicate that the B6 allele(s) of a gene(s) closely linked to *Mertk* functions as a suppressor of the photoreceptor degeneration.

### Retinal phenotype of *Mertk* ^*-/-*^ animals heterozygous for the suppressor

Intermixing of normal and abnormal retinal regions is an unusual phenotype, to our knowledge described for only one other naturally occurring inherited photoreceptor degeneration [30], with the exception of those subject to X-chromosome inactivation or associated with mitochondrial DNA mutations. We therefore performed a detailed histological analysis of the retinal phenotype of animals heterozygous for the recombinant chromosome. A vacuolated POS layer (Fig 1B) is an early and signal histological feature of photoreceptor degeneration due to loss of *Mertk* function in rodents [17]. Scoring this feature, we measured the size and location of degenerating and normal-appearing regions in single coronal sections from 20 *Mertk* ^*-/-*^ animals heterozygous for the recombinant chromosome. The peripheral retina exhibited degeneration in all cases, with relative preservation of the central (posterior) retina. The size of the degenerating peripheral region was greater in the dorsal (superior) as compared to ventral (inferior) retina (*P* = 1 x 10^−6^ by a two-tailed *t*-test). In addition to involvement of the periphery, 17 of 20 animals had one to four islands of degeneration bounded on both sides by normal-appearing retina. Evaluation of both eyes from a different group of 30 *Mertk* ^*-/-*^ mice heterozygous for the recombinant chromosome (16 females and 14 males) at ages ranging from P72 to P385 revealed a clear sex difference; females were more severely affected than males (means ± SEM of 39 ± 7.0% and 69 ± 3.8% normal retina for females and males, respectively; *P* = 0.0014 by a two-tailed *t*-test).

### Genetic mapping implicates *Tyro3* as a candidate modifier

**Fig 3:**
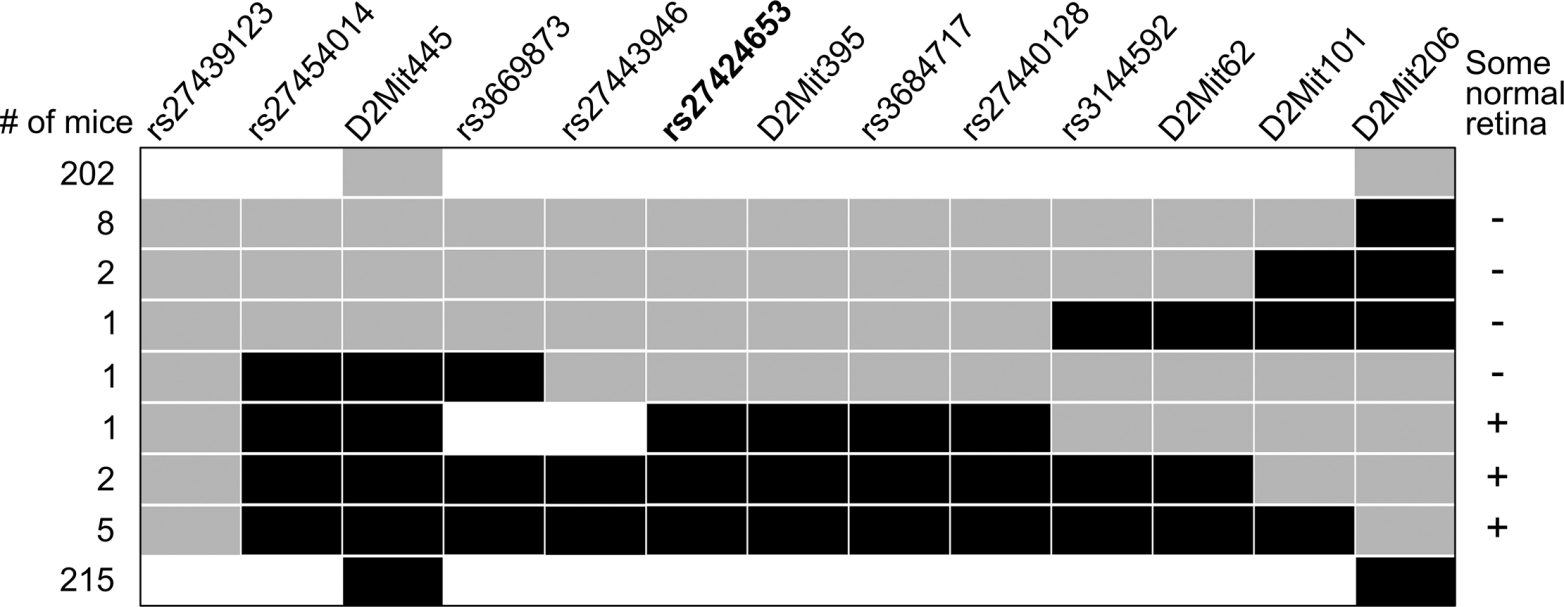
Meiotic mapping identifies *Tyro3* as a candidate modifier. Haplotype map of chromosomes (rows) from 437 offspring of a backcross. Only chromosomes originating from a heterozygous parent are depicted. Black indicates a B6 allele, gray a 129 allele, and white an unscored marker. Retinal phenotyping (at right) localized the modifier between rs3669873 and rs3144592, a region of 2,099,167 bp (GRCm38 assembly). Marker rs27424653 (bold) is in an intron of *Tyro3*, which lies completely within and near the middle of the critical interval.

We refined the location of the linked modifier through a backcross mapping approach. We genotyped 437 offspring, identified 20 recombinant chromosomes, and carried out histological analyses on retinas from the corresponding animals at 2 months of age, when the heterozygous suppressor phenotype can be readily distinguished from pan-retinal degeneration. We mapped the modifier to a critical interval of 2.1 megabases (Fig 3), a region that harbors 53 known or predicted protein coding genes (S1 Table). A literature search revealed that one of these genes is associated with phagocytosis–*Tyro3* [25], a paralog of *Mertk*.

### A cis-eQTL modulates *Tyro3* levels in the RPE

**Fig 4:**
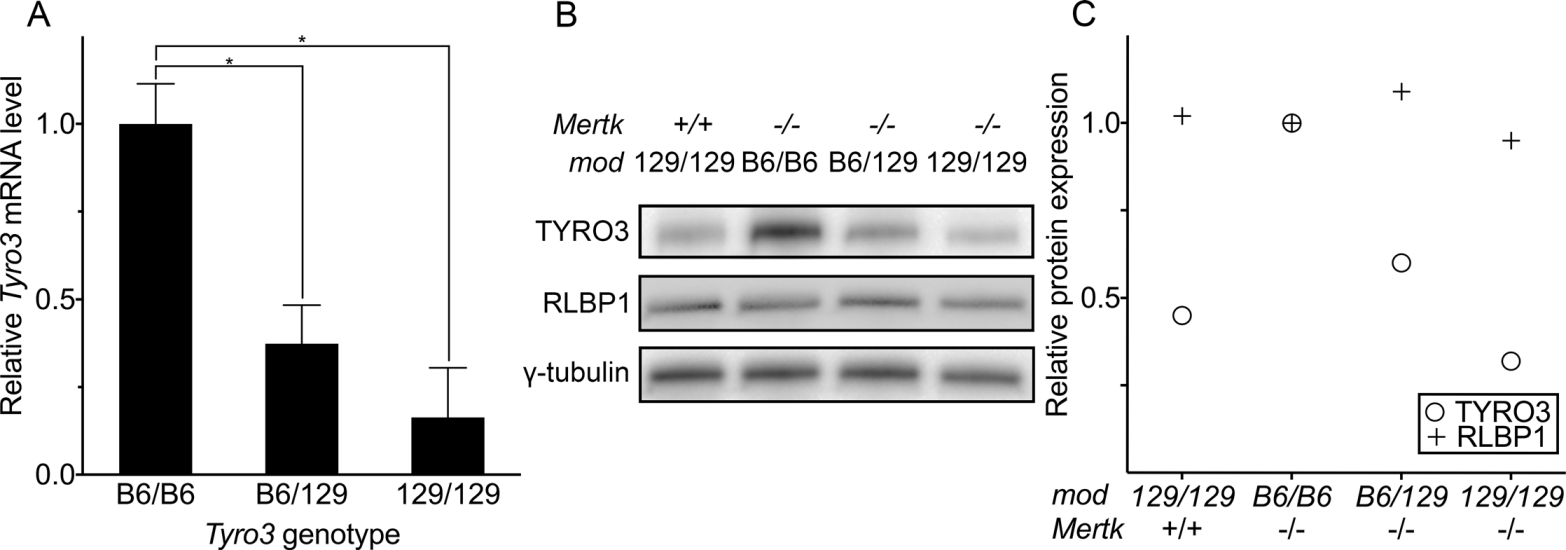
*Tyro3* mRNA and protein levels vary as a function of modifier genotype. (A) Quantitative RT-PCR of *Tyro3* mRNA from the RPE of incipient B6 congenic *Mertk* ^*-/-*^ mice with different modifier genotypes. For each line, individual samples from two P36 males were assayed in triplicate. The normalized means ± SD are depicted. * *P* ≤ 0.05 by one-way ANOVA with Bonferroni’s correction for multiple comparisons. (B) An immunoblot shows variation in the level of TYRO3 protein among the same three incipient B6 congenic *Mertk* ^*-/-*^ lines plus a fourth non-congenic *Mertk* ^*+/+*^ line homozygous for a 129 allele in the modifier region (leftmost lane). For each genotype, protein was isolated from eyecups pooled from two males, ages P40-45. (C) Quantification of the band intensities from (B) reveals a nearly linear relationship between genotypes and TYRO3 protein levels among the three lines, indicative of an expression quantitative trait locus (eQTL). TYRO3 and RLBP1 data were normalized to y-tubulin.

Prasad and colleagues demonstrated that *Mertk* ^*-/-*^ mice express a lower level of retinal *Tyro3* mRNA and protein compared to an unspecified wild-type control [26], but provided no explanation for this observation. If the *Tyro3* ^*129*^ allele is expressed at a lower level than its counterpart in B6, a strain commonly used as a wild-type control, co-inheritance of *Tyro3* ^*129*^ linked to the *Mertk* knockout allele could explain these earlier findings. We used quantitative RT-PCR to assess *Tyro3* mRNA levels in RPE isolated from B6 incipient congenic *Mertk* ^*-/-*^ mice that differ at the modifier region. The relative levels are *Tyro3* ^*B6/B6*^ > *Tyro3* ^*B6/129*^ > *Tyro3* ^*129/129*^ (Fig 4A). TYRO3 protein shows a similar modifier-allele-dependent gradation of levels in eyecups (RPE/choroid/sclera) (Fig 4B). Importantly, mice with the genotype *Mertk* ^*+/+*^ *;Tyro3* ^*129/129*^ exhibit a TYRO3 level comparable to that of *Mertk* ^*-/-*^ *;Tyro3* ^*129/129*^ mice, demonstrating that lower expression is a property of the *Tyro3* ^*129*^ allele and not secondary to retinal degeneration. We estimate that *Tyro3* ^*129/129*^ mice have about one-third the steady state level of *TYRO3* protein of incipient congenic *Tyro3* ^*B6/B6*^ animals (Fig 4C), similar to the previously described difference between “wild-type” and *Mertk* ^*-/-*^ animals [26]. The nearly additive relationship among *Tyro3* genotypes and expression levels, and the location of the responsible genetic variant(s) in the vicinity of the affected gene, are indicative of a local, or cis-eQTL.

The cis-eQTL we have identified influences RPE expression of *Tyro3* mRNA and protein, the levels of which correspond to the relative degree of photoreceptor degeneration observed for the three genotypes. Comparison of the *Tyro3* coding sequence between B6 and 129 revealed only a single amino acid difference: valine in B6 and leucine in 129 at position 811. This difference is predicted to be “benign” by PolyPhen-2 [31] (score = 0.000) and “tolerated” by SIFT [32]. Thus, any phenotypic differences associated with *Tyro3* B6 and 129 alleles likely derive from variation in the expression of the gene.

### *Tyro3* gene dosage modifies *Mertk*-associated photoreceptor degeneration

**Fig 5:**
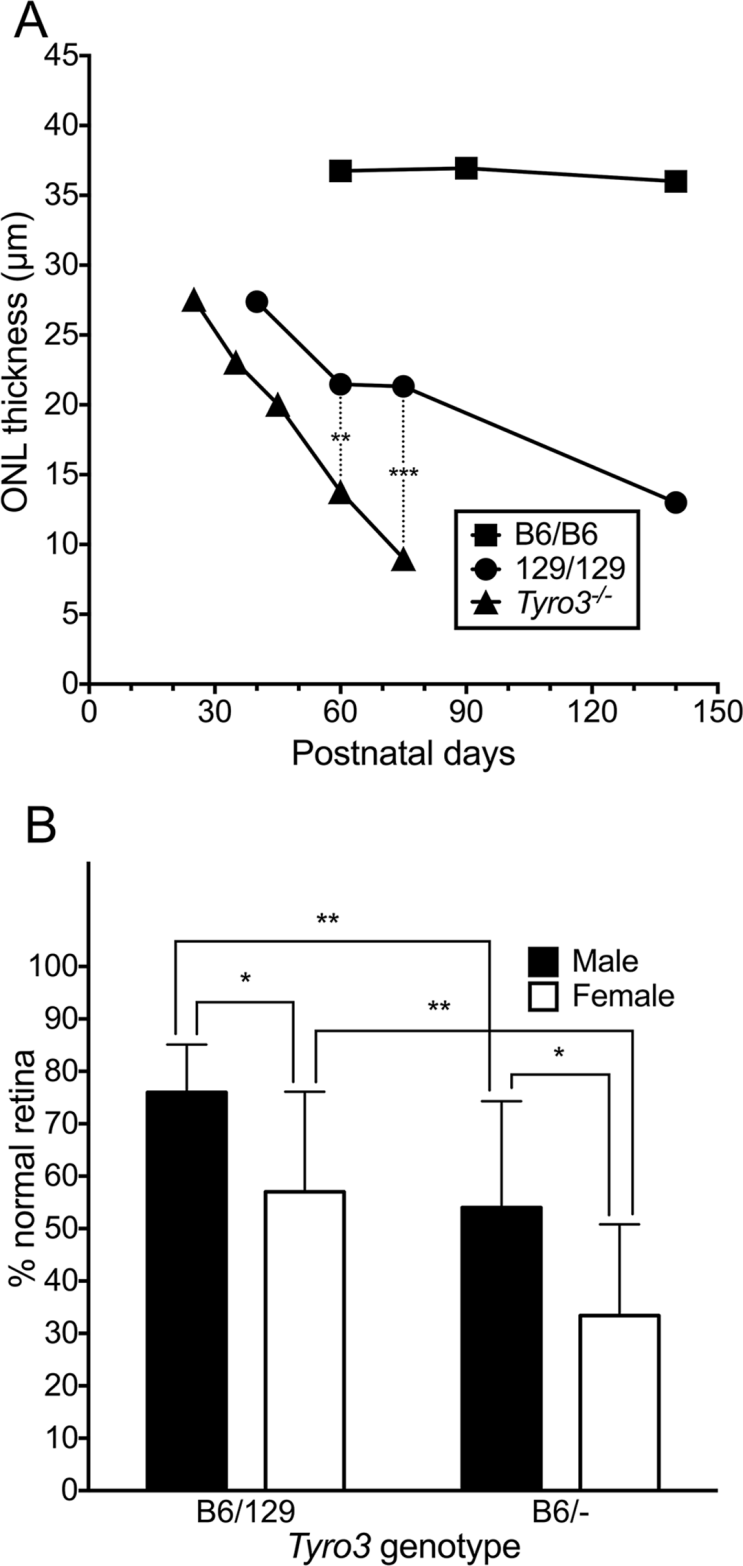
Genetic ablation of *Tyro3* accelerates photoreceptor degeneration in *Mertk* ^*-/-*^ mice. (A) Mean outer nuclear layer (ONL) thickness at various ages is shown for incipient B6 congenic *Mertk* ^*-/-*^ mice homozygous for either B6 or 129 modifier alleles, and also for *Mertk* ^*-/-*^ mice homozygous for a *Tyro3* knockout allele and 129 alleles at all other candidate modifier genes (*Tyro3* ^*-/-*^). Multiple animals were measured at each age, including three per line at P60 and five per line at P75 for the statistical comparisons. ** *P* = 0.0046 and *** *P* = 0.00014, both by two-tailed *t*-tests. (B) *Mertk* ^*-/-*^;*Tyro3* ^*B6/-*^ mice have more severe degeneration than *Mertk* ^*-/-*^;*Tyro3* ^*B6/129*^ animals, and females are more severely affected for both genotypes. Mean percentages ± SD of normal and degenerating retinal regions were obtained from both eyes of 54 offspring taken at P72-75 of a *Mertk* ^*-/-*^;*Tyro3* ^*B6/B6*^ x *Mertk* ^*-/-*^;*Tyro3* ^*129/-*^ cross. * *P* ≤ 0.05 and ** *P* ≤ 0.01 by a two-way ANOVA with Bonferroni’s correction for multiple comparisons.

If the level of *Tyro3* modifies *Mertk*-associated retinal degeneration, mice homozygous for both *Mertk* and *Tyro3* knockout alleles should display more rapid photoreceptor degeneration than those lacking *Mertk* function alone. *Mertk* ^*-/-*^ *;Tyro3* ^*-/-*^ mice do indeed exhibit more rapid photoreceptor degeneration than *Mertk* ^*-/-*^ *;Tyro3* ^*129/129*^ mice (Fig 5A). The modifier region in *Mertk* ^*-/-*^ *;Tyro3* ^*-/-*^ mice is of 129 origin (S2C Fig). Thus, of the candidate genes in the critical interval, only variation in *Tyro3* function can account for the phenotypic differences between *Mertk* ^*-/-*^ *;Tyro3* ^*129/129*^ and *Mertk* ^*-/-*^ *;Tyro3* ^*-/-*^ animals. These findings provide loss-of-function evidence for *Tyro3* as a genetic modifier of *Mertk*-associated retinal degeneration.

As an additional genetic test of the putative role of *Tyro3* as a modifier, we assessed the phenotype of *Mertk* ^*-/-*^ animals with one *Tyro3* ^*B6*^ allele and either a *Tyro3* ^*129*^ or *Tyro3* ^*-*^ (knockout) second allele. *Tyro3* ^*B6/-*^ animals should have less function than *Tyro3* ^B6/129^ animals and may exhibit a more severe retinal degeneration phenotype. We compared the retinas of 31 *Mertk* ^*-/-*^ *;Tyro3* ^B6/129^ animals to those of 23 *Mertk* ^*-/-*^ *;Tyro3* ^*B6/-*^ mice, all derived from a single *Mertk* ^*-/-*^ *;Tyro3* ^B6/B6^ male crossed with three *Mertk* ^*-/-*^ *;Tyro3* ^129/-^ sisters. Both male and female *Mertk* ^*-/-*^ *;Tyro3* ^B6/-^ offspring exhibited significantly more retinal degeneration than sex-matched *Mertk* ^*-/-*^ *;Tyro3* ^B6/129^ animals (Fig 5B). For both genotypes, females were again more severely affected than males. Recalling the observation that *Tyro3* ^*129/129*^ animals exhibit ~33% the level of expression of *Tyro3* ^B6/B6^ mice, these results indicate that *Mertk*-deficient retinas are exquisitely sensitive to *Tyro3* function (~67% for *Tyro3* ^B6/129^ vs. ~50% for *Tyro3* ^B6/-^, with *Tyro3* ^*B6/B6*^ assigned as 100%).

**Fig 6:**
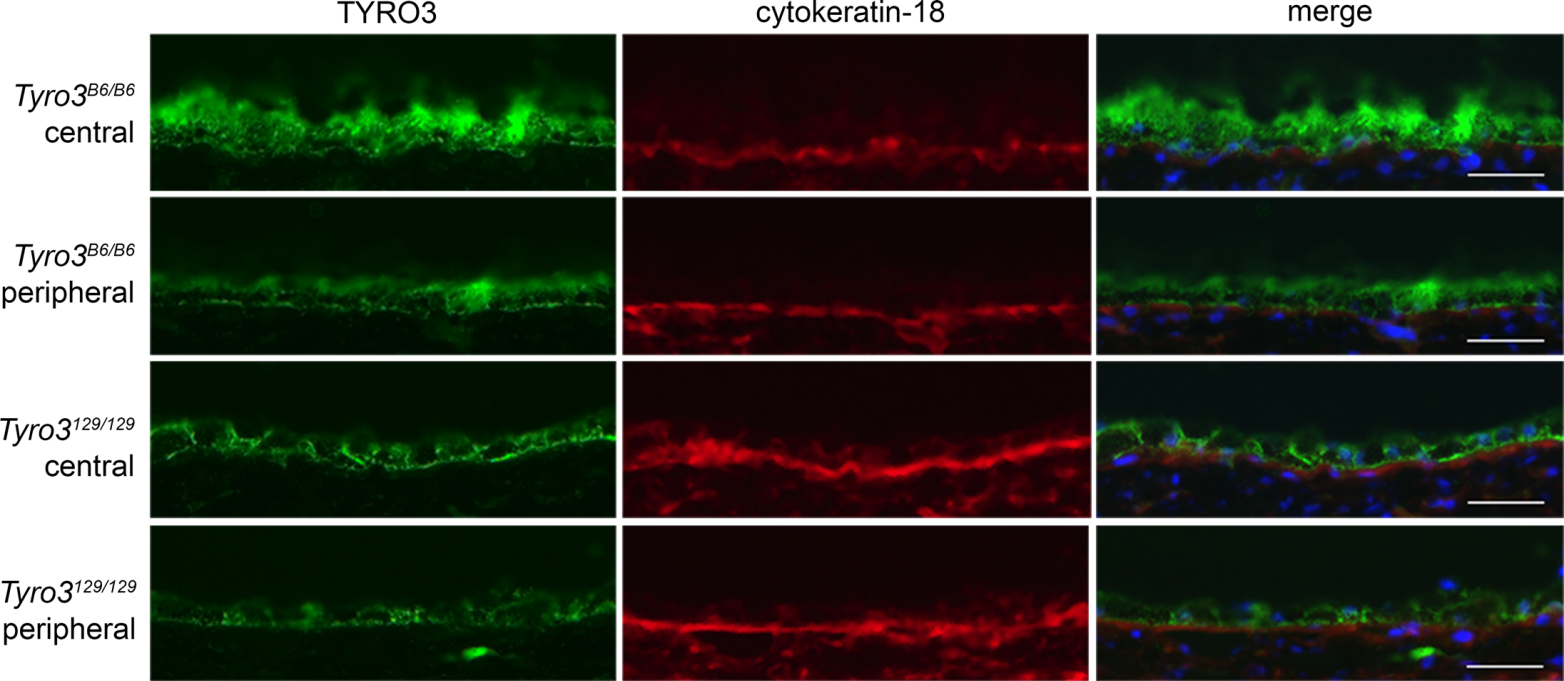
A central to peripheral gradient of TYRO3 expression correlates with regional variation in retinal preservation. Immunostaining of frozen sections from P250 animals demonstrates higher expression of TYRO3 protein in the central RPE of a *Mertk* ^*-/-*^;*Tyro3* ^*B6/B6*^ mouse compared to the periphery. The central retina of *Mertk* ^*-/-*^;*Tyro3* ^*B6/B6*^ animals escapes degeneration, while the other areas pictured do not. As a control, the same sections were stained for the RPE-expressed protein, cytokeratin-18. Apical TYRO3 staining is evident in both regions of the B6 retina, whereas such staining is not apparent in 129. Scale bar = 20 μm.

Our findings suggest that the *Tyro3* ^*129*^ allele is hypomorphic in the RPE and that variation in retinal TYRO3 protein function modulates the severity of *Mertk*-associated degeneration. In support of this concept, TYRO3 is expressed at higher levels in the preserved central retinas of *Mertk* ^*-/-*^;*Tyro3* ^*B6/B6*^ animals than in their degenerating peripheral retinas, where levels appear similar to those seen in the central retinas of *Mertk* ^*-/-*^;*Tyro3* ^*129/129*^ mice (Fig 6).

### TYRO3 protein co-localizes with phagosomes and promotes phagocytosis

**Fig 7:**
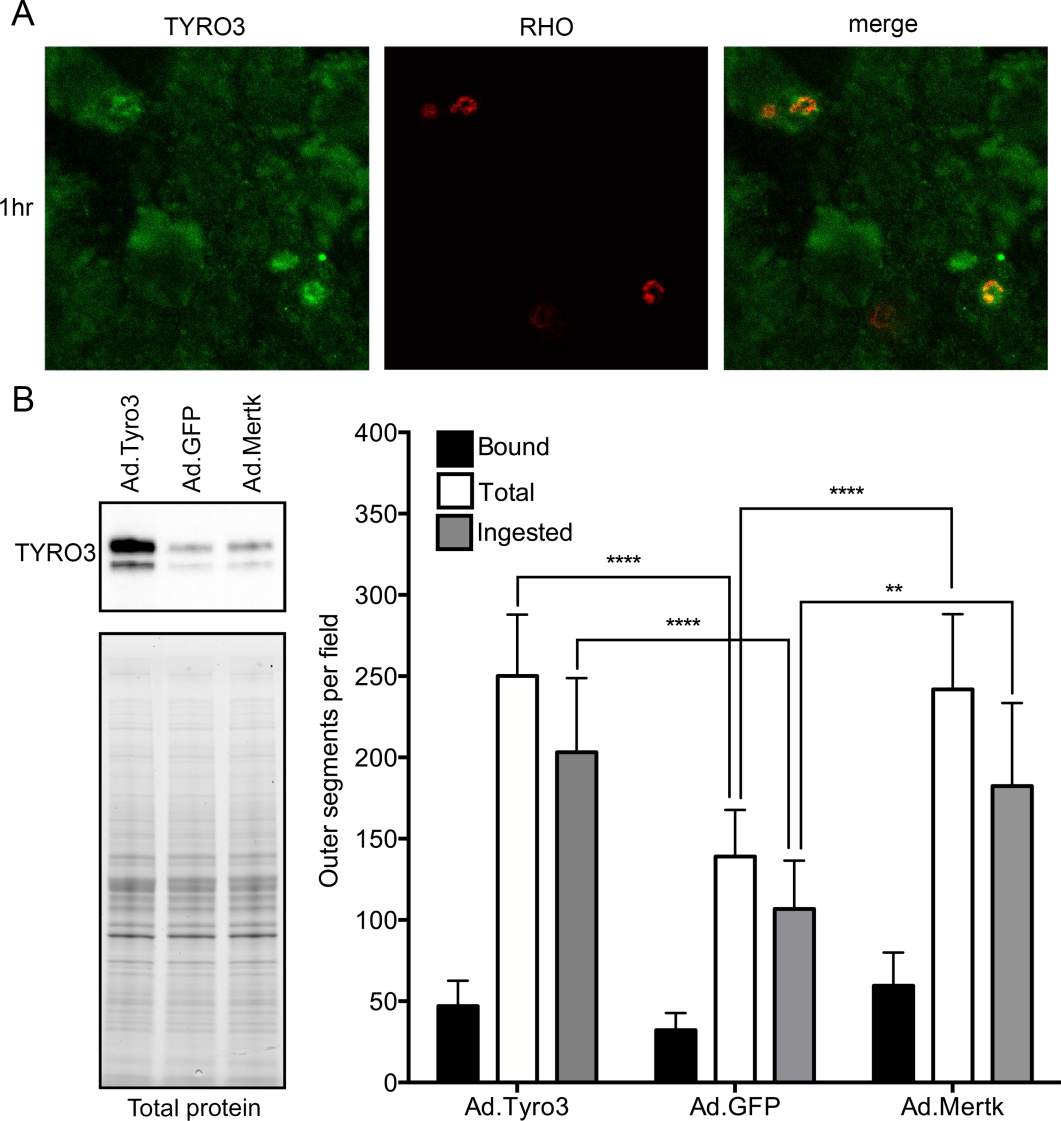
TYRO3 localizes to nascent phagosomes and promotes photoreceptor outer segment (POS) phagocytosis. (A) Confocal microscopy of primary human RPE cells challenged with bovine POS demonstrates co-localization of TYRO3 (green) with rhodopsin (red) after 1 hour. (B) Adenoviral vector-mediated overexpression of murine TYRO3 in NRK-49F cells stimulates POS phagocytosis, in particular POS ingestion (total minus bound). Adenoviral vectors encoding GFP and MERTK were used as negative and positive controls, respectively. Means ± SD are depicted. **** *P* ≤ 0.0001, ** *P* ≤ 0.01 by a two-way ANOVA with Bonferroni’s correction for multiple comparisons.

Our demonstration of increased RPE phagosomes in normal-appearing areas of *Mertk* ^*-/-*^ *;Tyro3* ^*B6/129*^ retinas (Fig 1D) suggested that TYRO3 may function in RPE phagocytosis. We used confocal immunofluorescence microscopy to assess the possible co-localization of TYRO3 with nascent phagosomes of bovine POS ingested by human primary RPE cells, which express endogenous TYRO3 (S3A Fig). Images taken over a time course revealed initial co-localization of TYRO3 with rhodopsin-positive phagosomes 1–2 hours after POS addition (Fig 7A; S3B Fig), and diminished co-localization after three hours (S3B Fig). MERTK shows similar co-localization in cultured rat primary RPE cells challenged with POS [33]. As a gain-of-function test of the role of TYRO3 in POS phagocytosis, we expressed murine TYRO3 in normal rat kidney fibroblasts (NRK-49F), which phagocytize POS following overexpression of rat MERTK [34]. Expression of mTYRO3 also stimulates ingestion of POS by NRK-49F cells (Fig 7B). We obtained similar results following expression of mTYRO3 in primary RPE cells from *Mertk* ^*-/-*^ *;Tyro3* ^*-/-*^ mice (S4 Fig). Together, our data indicate that *Tyro3* modifies *Mertk*-associated retinal degeneration by modulating the phagocytic capability of the RPE.

## Discussion

Rats [18], mice [17] and humans [9] with loss of MERTK function exhibit rapid photoreceptor degeneration secondary to a profound defect in RPE phagocytosis of POS tips. We have discovered a genetic modifier of *Mertk*-associated retinal disease in mice, the B6 allele of which (in the homozygous state) almost completely preserves retinal structure far beyond the age at which photoreceptors degenerate in incipient congenic animals homozygous for the 129 allele. This striking suppression of degeneration is accompanied by an increase in RPE phagocytosis in areas of retinal preservation (Fig 1D). Meiotic mapping localized the modifier to a region encoding 53 candidate genes, including the *Mertk* paralog, *Tyro3* (Fig 3). *Tyro3* is known to be expressed in the murine RPE [26], but it’s role there was ill-defined. We hypothesized that variation in *Tyro3* function accounts for most or all of the modifier effect. We have obtained substantial evidence in support of this hypothesis. We demonstrate that *Tyro3* gene dosage modulates the severity of *Mertk*-associated retinal degeneration (Fig 5). We further show that the *Tyro3* ^*B6*^ allele is expressed in the RPE at a three-fold higher level, compared to *Tyro3* ^*129*^ (Fig 4), engendering the inference that, just as diminished *Tyro3* function exacerbates *Mertk*-associated degeneration (Fig 5), increased *Tyro3* function explains the protective effect seen in *Mertk-/-;Tyro3* ^*B6/B6*^ animals. Consistent with this inference, higher TYRO3 protein expression correlates with retinal preservation in *Mertk-/-;Tyro3* ^*B6/B6*^ mice (Fig 6). Finally, we provide a mechanistic explanation for restoration of RPE phagocytic activity in B6 modifier mice by demonstrating that expression of TYRO3 stimulates phagocytic ingestion of POS in cultured murine RPE cells (S4 Fig) and rat kidney fibroblasts (Fig 7B), and that endogenous TYRO3 co-localizes with sites of POS ingestion in primary human RPE cells (Fig 7A; S3B Fig). While we cannot rule out the contribution of other critical interval genes to the modifier effect, our results provide strong evidence that TYRO3 functions directly in RPE phagocytosis of POS, and suggest that, at sufficient levels of expression, TYRO3 can effectively compensate for loss of MERTK function and protect the retina from degeneration.

Our findings are consistent with the slow peripheral retinal degeneration observed in mice with a *Mertk* missense mutation generated by ENU mutagenesis on a B6 background [35], and indicate that a widely available line of *Mertk* knockout mice is hypomorphic for *Tyro3* due to reduced expression from a tightly linked *Tyro3* ^*129*^ allele. Thus, both TYRO3 and MERTK likely function to maintain retinal homeostasis in mice by enabling RPE phagocytosis of POS tips, and the conclusion that MERTK plays a predominant role in the process [27] should be revised. TYRO3 and MERTK may act during RPE phagocytosis by a similar mechanism. Like TYRO3, MERTK localizes to nascent phagosomes [33], both receptors enhance the ingestion phase of the process (Fig 7B, S4 Fig and [33]), and both are activated by the ligands PROS1 and GAS6 [26,27] present in the outer retina. TYRO3 does not appear to require another TAM receptor to promote RPE phagocytosis in vivo; the *Mertk* knockout allele does not produce detectable protein [17], and *Axl* is not expressed at an appreciable level in the murine RPE [36]. Whether there are differences between MERTK and TYRO3 in, for example, the kinetics of receptor localization and/or activation during RPE phagocytosis, remains to be investigated.

Our work may have implications beyond photoreceptor degeneration. Several groups have studied the immune and fertility phenotypes of single and/or double TAM receptor knockout mice in order to draw conclusions regarding the role of individual TAM receptors in these processes. Assessments made on the basis of mice homozygous for only a *Mertk* knockout allele may obscure the importance of *Tyro3* because of lower expression from the linked 129 allele. For example, the conclusions that clearance of apoptotic thymocytes [25] and postpartum mammary gland efferocytosis [22] require *Mertk*, but not *Tyro3*, may need to be re-examined. Similarly, reduction in *Tyro3* expression may contribute to male infertility [37] and the response to retinal self antigen [38] of *Axl* and *Mertk* double-knockout mice.

The intermixing of degenerating and normal regions we observed in *Mertk* ^*-/-*^ *;Tyro3* ^*B6/129*^ animals is an unusual feature among naturally occurring murine models of retinal degeneration. In light of the increased numbers of RPE phagosomes in normal regions of *Mertk* ^*-/-*^ *;Tyro3* ^*B6/129*^ mice (Fig 1D), the correspondence of TYRO3 levels with the pattern of sparing and degeneration in *Mertk* ^*-/-*^ *;Tyro3* ^*B6/B6*^ animals (Fig 6), and our data indicating a direct role for TYRO3 in POS phagocytosis (Fig 7; S3 and S4 Figs), we posit that epigenetic variation in *Tyro3* levels in RPE cells causes the intermixing phenotype. The decrease in percentage of preserved normal retina in *Mertk* ^*-/-*^ mice across a *Tyro3* allelic series, *Tyro3* ^*B6/B6*^ > *Tyro3* ^*B6/129*^ > *Tyro3* ^*B6/-*^, until it reaches zero in *Tyro3* ^*129/129*^ and *Tyro3* ^*-/-*^ animals, suggests fluctuations above and below a critical threshold level necessary for photoreceptor viability. Our estimate that differences of ~20% in average expression levels between *Tyro3* ^*B6/129*^ and *Tyro3* ^*B6/-*^ mice can modulate the phenotype suggests that the magnitude of such fluctuations need not be large. Stochastic fluctuations in gene expression have recently been implicated in zebrafish retinal ganglion cell development [39,40], and have been invoked to explain changes in the aging RPE [41,42]. Our results suggest that stochastic processes during RPE development can also have phenotypic consequences.

One or more naturally occurring, cis-acting DNA sequence variants modulate *Tyro3* expression. Comparison of 144 single nucleotide polymorphisms among 15 re-sequenced mouse strains (S2 Table) shows that the *Tyro3* B6 haplotype is almost identical to MOLF/EiJ, very similar to PWK/Phj, and very different from 12 other common laboratory strains including 129P2/Ola. Both MOLF/EiJ and PWK/Phj are non-*domesticus* wild-derived, emphasizing the atypical nature of this modifier allele among inbred strains, which on average are 92% *mus musculus domesticus* in origin [43]. Modifiers arising from wild-derived variants should be shaped by natural selection and may correspond to nodes in genetic networks with greater intrinsic plasticity [44]. *Tyro3* appears to be a modifier in the strict sense of the term [44] because variation in its function alone does not have an obvious retinal phenotype [25]. Our findings illustrate that, in the context of a recessive IPD, the phenotypic consequences of a naturally occurring eQTL variant can be unmasked.

Almost all currently characterized modifiers of IPDs are coding sequence variants. Our results suggest that eQTL may contribute to phenotypic variability in human IPDs. Additional examples of eQTL-associated IPD modifiers may await discovery because more than 200 genes are associated with human IPDs, a significant fraction of eQTL are cell-type-restricted [45,46], and eQTL in human ocular tissues have not been assessed. Increased *TYRO3* levels in the RPE may underlie the slower pace of photoreceptor degeneration described for some individuals with *MERTK* mutations [11], and may contribute to the low frequency of *MERTK* mutations observed among RP cohorts sampled from certain ethnic populations [47,48]. Our work provides a rationale for a comprehensive search for eQTL in human ocular tissues and, in particular, the RPE.

## Methods

### Ethics statement

All procedures were in compliance with the Association for Research in Vision and Ophthalmology Statement for the Use of Animals, and were approved by the Stanford University Administrative Panel on Laboratory Animal Care (APLAC) and the University of California, San Francisco IACUC. Mice were anesthetized with ketamine and xylazine, and euthanized by a regulated flow of carbon dioxide gas.

### Animals

All procedures were in compliance with the Association for Research in Vision and Ophthalmology Statement for the use of Animals, and were approved by the Stanford University Administrative Panel on Laboratory Animal Care (APLAC) and the University of California, San Francisco IACUC. We obtained mice homozygous for a knockout allele of *Mertk* [28] from Dr. Glenn Matsushima (University of North Carolina). After characterizing the retinal phenotype of these mice [17], which were described as crossed four times to B6, we performed six additional generations of backcrossing. Animals heterozygous for a recombinant chromosome were identified at this stage and incipient congenic lines homozygous for B6 or 129 modifier alleles were generated by crossing heterozygous animals. Albino lines homozygous for each modifier allele were generated by crossing each pigmented homozygous line with C57BL/6-c(2J) (JAX stock number 000058) and intercrossing the F1 offspring. Subsequent modifier heterozygotes were generated by intercrossing the respective pigmented or albino homozygous lines. Mice homozygous for knockout alleles of both *Mertk* and *Tyro3* were generously provided by Dr. Stephen Goff (Columbia University) and subsequently backcrossed to B6 at least four times. Genotyping of selected animals of various *Tyro3* genotypes in our colony showed them to be negative for the *rd8* [49] and *rd1* [50] mutations and to be homozygous for the 450Met allele of *Rpe65* [51].

### Genotyping

DNA was isolated from tail snips by standard methods. STR markers were scored by agarose gel electrophoresis. Single nucleotide polymorphism (SNP) markers were scored by dideoxynucleotide sequencing. S3 Table lists oligonucleotide sequences for the markers used, and S4 Table lists the genome coordinates of these markers, along with those of *Mertk* and *Tyro3*.

### Meiotic mapping

We crossed *Mertk* ^*-/-*^ animals heterozygous for B6 and 129 alleles in the modifier region (S2B Fig) with incipient congenic *Mertk* ^*-/-*^ animals homozygous for 129 alleles in the region (S2A Fig), and genotyped 437 offspring with markers D2Mit206 and D2Mit445. DNA samples containing recombinant chromosomes were genotyped with additional STR and SNP markers to more precisely map recombination breakpoints. The eyes of animals with recombinant chromosomes were taken at about P70 and processed for histology to assess the presence or absence of any normal-appearing retina. Retinal histology was not done for animals with parental genotypes. Nonrecombinant phenotypes were inferred from histological analysis of 99 other *Mertk* ^*-/-*^ animals heterozygous for the modifier region (i.e., 129/B6), all of which had some normal retina at ages ranging from P59 to > P500, and of 66 *Mertk* ^*-/-*^ mice homozygous for the 129 modifier allele, none of which exhibited any normal retina at ages ranging from P59 to P240.

### Histology

Enucleated eyes were fixed by immersion in a mixed aldehyde solution, epoxy-embedded, sectioned along the vertical meridian at 1 μm thickness and stained as previously described [52]. Quantification of outer nuclear layer thickness was done as previously described [52]. Phagosomes in both the RPE cell processes and cell bodies were identified using previously described criteria [17] and counted in 240 μm segments across the full length of a single coronal section from eyes taken 1 hour after light onset in entrained animals. Degenerating regions were identified by the presence of vacuolated POS [17]. To assess the effect of *Tyro3* gene dosage, we measured the fraction of normal-appearing retina across the entire arc of a single coronal section for each animal.

### Electroretinography

Mice were dark-adapted overnight and anesthetized with ketamine (80mg/kg) and xylazine (13mg/kg). Eyes were dilated with 1% atropine, 2.5% phenylephrine hydrochloride, and 0.5% propracaine hydrochloride, and mice were placed in a Ganzfeld Color Dome controlled by a signal averaging system (Espion). ERGs were recorded from both eyes using lens electrodes placed on the cornea with 2.5% hypromellose. A ground electrode was placed in the tail and a reference electrode in the nose, and mice were kept on a heating pad. Dark-adapted (scotopic) stimuli consisted of eight flashes increasing in intensity from 0.001 to 10 cd • s/m^2^. Five trials were averaged for each dark-adapted flash intensity. For light-adapted (photopic) readings, rod responses were first saturated by exposing mice to 20 cd • s/m^2^ for 10 minutes, and stimuli were then presented as six flashes increasing in intensity from 0.78 to 20 cd • s/m^2^. Twenty-five trials were averaged for each light-adapted flash intensity. Data from both eyes were averaged.

### Quantification of Tyro3 in mouse eyes

We used a published method [53] to isolate RPE RNA, and carried out quantitative RT-PCR as previously described [54] using primers listed in S3 Table. Protein lysates from eyecups (RPE/choroid/sclera) were prepared as previously described [54]. Protein transfer and chemiluminescence detection for immunoblot analysis for TYRO3, RLBP1, and y-tubulin (S5 Table) were done as described previously [55]. The intensity of bands was quantified by ImageJ and normalized to y-tubulin.

### *Tyro3* cDNA sequence

Whole genome sequence for the 129P2/Ola strain is available through the Sanger Institute’s Mouse Genomes Project http://www.sanger.ac.uk/resources/mouse/genomes/. Comparison of the *Tyro3* coding sequence between this strain and the B6 reference genome revealed a number of synonymous substitutions and a single non-synonymous change, as described in Results. We isolated RNA from RPE cells [53] of *Mertk* ^*-/-*^ *;Tyro3* ^*129/129*^ mice, synthesized cDNA, and sequenced overlapping PCR products generated using primer pairs listed in S3 Table. The *Tyro3* coding sequence from these mice contained all of the substitutions expected from the Sanger Institute’s genome sequence and no others.

### Immunofluorescence microscopy

Cryosections were prepared as previously described [54], permeabilized with 3% normal goat serum/0.3% Triton X-100/1X PBS at room temperature for 30 minutes, and then blocked with 50% normal goat serum/50% antibody buffer (5% normal goat serum/0.1% Triton X-100/1X PBS) at room temperature for 1 hour. Sections were incubated with primary antibodies (S5 Table) overnight at 4°C, then either with a biotinylated anti-rabbit IgG secondary antibody for 20 minutes followed by fluorescein-conjugated avidin for TYRO3 detection, or with an Alexa 594-conjugated anti-mouse IgG secondary antibody for cytokeratin detection. Sections were stained with 4’, 6 diamidino-2-phenylindole (DAPI) for 10 minutes, and slides were mounted with ProLong Gold Antifade Reagent (Cell Signaling). Images were taken with the same exposure settings for all genotypes using a Zeiss Axiocam MRc5 microscope, and formatted with Photoshop CS6 (Adobe).

### Human primary RPE culture

Human fetal eyes were obtained from Advanced Bioscience Resources (Alameda, CA). RPE cells were isolated and cultured according to the methods of Maminishkis and Miller [56]. After the cells became pigmented, they were trypsinized and plated on 12-mm diameter transwells (Corning Costar) at an initial density of 150,000 cells/well. The cells were deemed ready for use once they were evenly pigmented and the background-subtracted trans-epithelial resistance was above 200 Ω.cm^2^.

### Confocal microscopy

Cultured human primary RPE cells were incubated for 1 to 3 hours with bovine POS, isolated as previously described [57]. Unbound POS were removed by washing with DMEM. Cells were fixed by 4% paraformaldehyde in PBS for 30 minutes and cell membranes were permeabilized with 0.1% Triton X-100. TYRO3 and POS were labeled with anti-TYRO3 and anti-rhodopsin antibodies (S5 Table), respectively, followed by Alexa Fluo488-conjugated anti-rabbit IgG and Texas Red-conjugated anti-mouse IgG. Slides were mounted with mounting medium (Vectashield) and viewed under a confocal microscope.

### Adenoviral transduction

Recombinant Ad-*Mertk* and Ad-*GFP* were as previously described [33]. Recombinant Ad-*Tyro3* containing a murine *Tyro3* open reading frame (NM_019392) under the control of a CMV promoter was constructed using the ViraPower Adenoviral Expression System (Life Technologies). A full coding *Tyro3* cDNA clone (IMAGE: 6834015) with a truncated 3’ untranslated region was ligated between the *Sal* I to *Kpn* I sites of pENTR11, subsequent to removal from the vector of the ccdB and CmR genes by *EcoR* I digestion and religation. The resulting plasmid was recombined with pAD/CMV/V5-DEST. Virions were purified by CsCl gradient centrifugation. The rat kidney fibroblast cell line NRK-49F was obtained from American Type Culture Collection and maintained as recommended by the vendor. NRK-49F cells were plated on 8-well chamber slides and cultured for 24 hours before transduction with recombinant adenoviruses. The next day, cells were used for phagocytosis assays or immunoblotting. Murine primary RPE cells were isolated and cultured as previously described [58] on 6.5 mm transwell inserts with a 0.4 μm pore size (Corning) for 7 days before transduction with recombinant adenoviruses. Cells were used for phagocytosis assays 3 days after transduction.

### Phagocytosis assay

Each well of adenovirus infected NRK-49F cells was challenged with 5 x 10^6^ bovine POS in culture medium containing 5% FBS. After 4 hours incubation, unbound POS were washed away 3 times with cold PBS on ice, and the cells were fixed with 4% paraformaldehyde for 30 minutes. To distinguish total and bound POS, samples were divided into two groups. Each group contained two wells of cells. Group 1 was permeabilized with 0.1% Triton X-100 and group 2 remained unpermeabilized. POS were immunolabeled with anti-rhodopsin monoclonal antibody rho 4D2 [59] (S5 Table), followed by a Texas Red-conjugated anti-mouse IgG. For adenovirus-transduced mouse RPE cells, each transwell insert was challenged with 5 x 10^6^ bovine POS in culture medium containing 5% FBS. After 3 hours incubation, unbound POS were washed away three times with cold PBS, and the cells were fixed with 4% paraformaldehyde for 30 minutes. Bound and total POS were differentially labeled as previously described [57] with anti-rhodopsin monoclonal antibody rho 4D2 [59] (S5 Table). RPE cell nuclei were labeled with DAPI (Invitrogen D1306) before mounting. A fluorescence microscope was used to collect images from arbitrary fields for each group. POS numbers on each image were quantified with ImageJ.

### Immunoblotting

Whole cell lysates of recombinant adenovirus transduced NRK-49F cells were separated on a 4–20% gradient Criterion Stain-Free TGX gel (Bio-Rad). Proteins were then transferred to a nitrocellulose membrane using a Trans-Blot Turbo transfer system (Bio-Rad). The stain-free gel and chemiluminescent blot images were captured using a ChemiDoc MP Imaging system (Bio-Rad) and quantified with Image Lab software (Bio-Rad) using total protein in each lane as a loading control to normalize signals from individual proteins. TYRO3 expression was probed by anti-TYRO3 (S5 Table) followed by horseradish peroxidase-conjugated secondary antibody and Clarity western ECL substrate (Bio-Rad).

## Supporting Information

S1 Fig Retinal preservation in aged *Mertk* ^*-/-*^ mice. A retinal section from a *Mertk* ^*-/-*^ mouse at P366 demonstrates an island of degeneration (middle of image), characterized by disorganized outer segments and a thinned outer nuclear layer, that is bounded on both sides by normal-appearing retina.
(DOCX)

S2 Fig Genotypes surrounding the *Mertk* knockout allele in three mouse lines. Black rectangles indicate homozygosity for B6 alleles. Gray rectangles indicate homozygosity for 129 alleles. Mixed rectangles indicate heterozygosity for B6 and 129 alleles. (A) In *Mertk* ^*-/-*^ animals with pan-retinal photoreceptor degeneration, a large segment of chromosome 2 remains homozygous for 129 alleles after more than six generations of backcrossing to C57BL/6 (B6). (B) Heterozygosity for 129 and B6 alleles in backcrossed *Mertk* ^*-/-*^ mice with areas of histologically normal retina provides evidence for a recombinant chromosome harboring a B6 suppressor allele. (C) *Mertk* ^*-/-*^ *;Tyro3* ^*-/-*^ mice are homozygous for 129 alleles throughout the modifier critical interval (approximated by a line) and beyond.
(PDF)

S3 Fig TYRO3 is expressed in cultured primary human RPE cells and co-localizes with POS in a phagocytic assay. (A) Immunoblot for TYRO3 in four differentiated human primary RPE cell lines. The stain-free gel and chemiluminescent blot images were captured using a ChemiDoc MP Imaging system (Bio-Rad). (B) Confocal images of primary human RPE cells from the same experiment as in Fig 7A show persistent, but diminished, co-localization of endogenous TYRO3 and bovine POS at later time points. White arrowheads mark sites of co-localization of TYRO3 and POS.
(TIF)

S4 Fig Expression of *Tyro3* promotes photoreceptor outer segment phagocytosis by *Mertk* ^*-/-*^ *;Tyro3* ^*-/-*^ RPE cells. Primary mouse RPE cells were cultured, transduced with an adenoviral vector encoding murine *Tyro3* or rat *Mertk*, and then assayed for their ability to phagocytize bovine outer segments (OS). Means ± SD are depicted. *Tyro3* expression stimulates OS ingestion (total minus bound) compared to a no-virus control, as does rat *Mertk*. *** *P* ≤ 0.001, ** *P* ≤ 0.01, * *P* ≤ 0.05, calculated by two-way ANOVA with Bonferroni’s correction for multiple comparisons.
(TIFF)

S1 Table Protein coding genes within the modifier critical interval. (PDF)

S2 Table *Tyro3* haplotypes. (PDF)

S3 Table Oligonucleotides used. (PDF)

S4 Table Gene and marker genome coordinates. (PDF)

S5 Table Antibodies used. (PDF)
