# Supplementary figures and images for: Tyro3 Modulates Mertk-Associated Retinal Degeneration
Source: PLoS Genet. 2015 Dec 11;11(12):e1005723. doi: 10.1371/journal.pgen.1005723 (PMC4687644; doi:10.1371/journal.pgen.1005723)

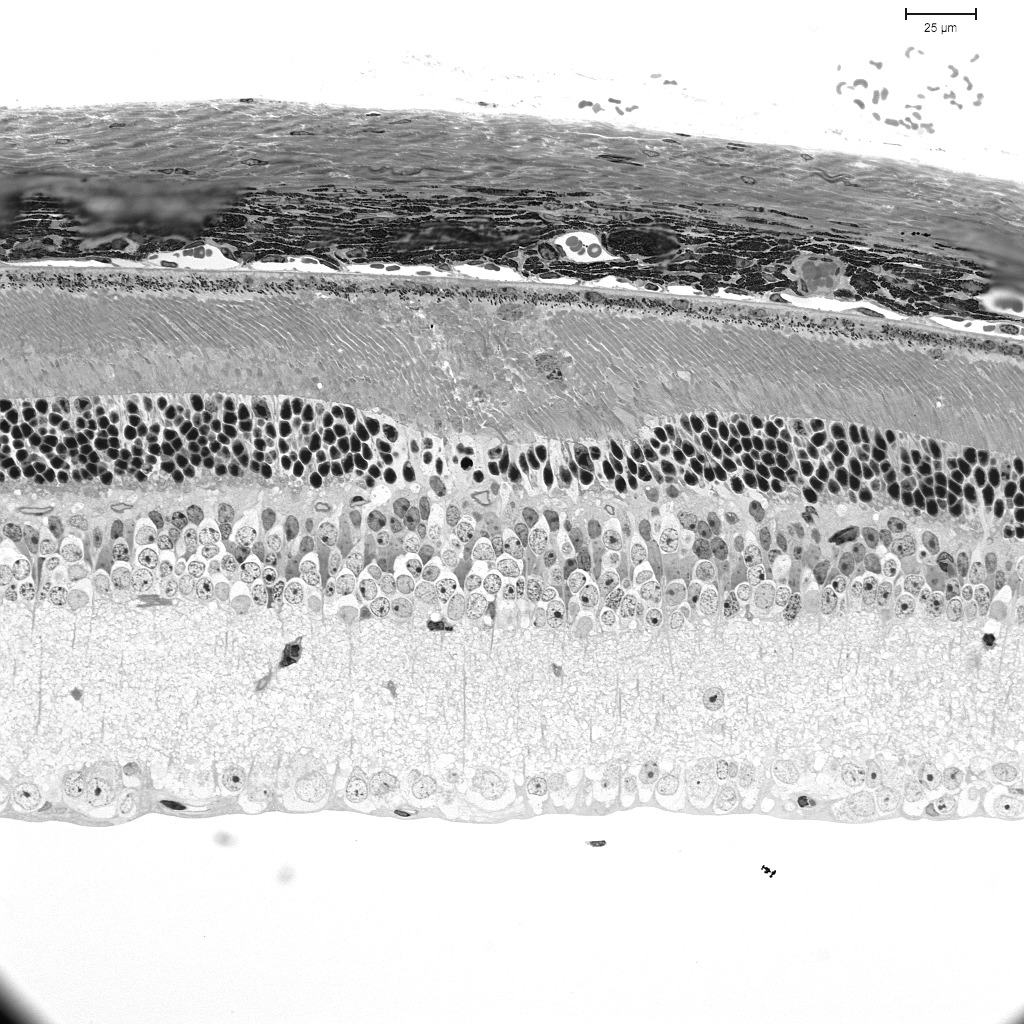
**S1 Figure**

Supplement: S1 Fig — A retinal section from a Mertk -/- mouse at P366 demonstrates an island of degeneration (middle of image), characterized by disorganized outer segments and a thinned outer nuclear layer, that is bounded on both sides by normal-appearing retina. (DOCX) [file pgen.1005723.s001.docx]

S2 Figure

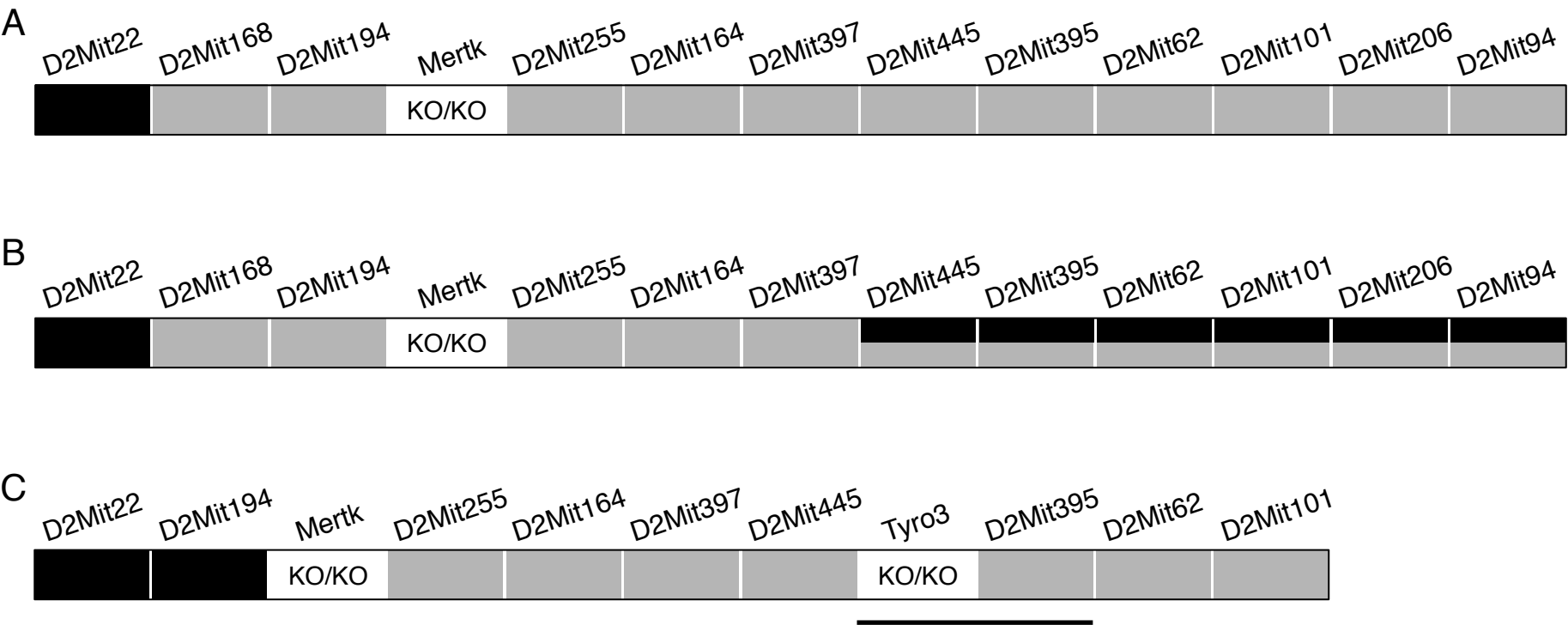

Supplement: S2 Fig — Black rectangles indicate homozygosity for B6 alleles. Gray rectangles indicate homozygosity for 129 alleles. Mixed rectangles indicate heterozygosity for B6 and 129 alleles. (A) In Mertk -/- animals with pan-retinal photoreceptor degeneration, a large segment of chromosome 2 remains homozygous for 129 alleles after more than six generations of backcrossing to C57BL/6 (B6). (B) Heterozygosity for 129 and B6 alleles in backcrossed Mertk -/- mice with areas of histologically normal retina provides evidence for a recombinant chromosome harboring a B6 suppressor allele. (C) Mertk -/- ;Tyro3 -/- mice are homozygous for 129 alleles throughout the modifier critical interval (approximated by a line) and beyond. (PDF) [file pgen.1005723.s002.pdf]

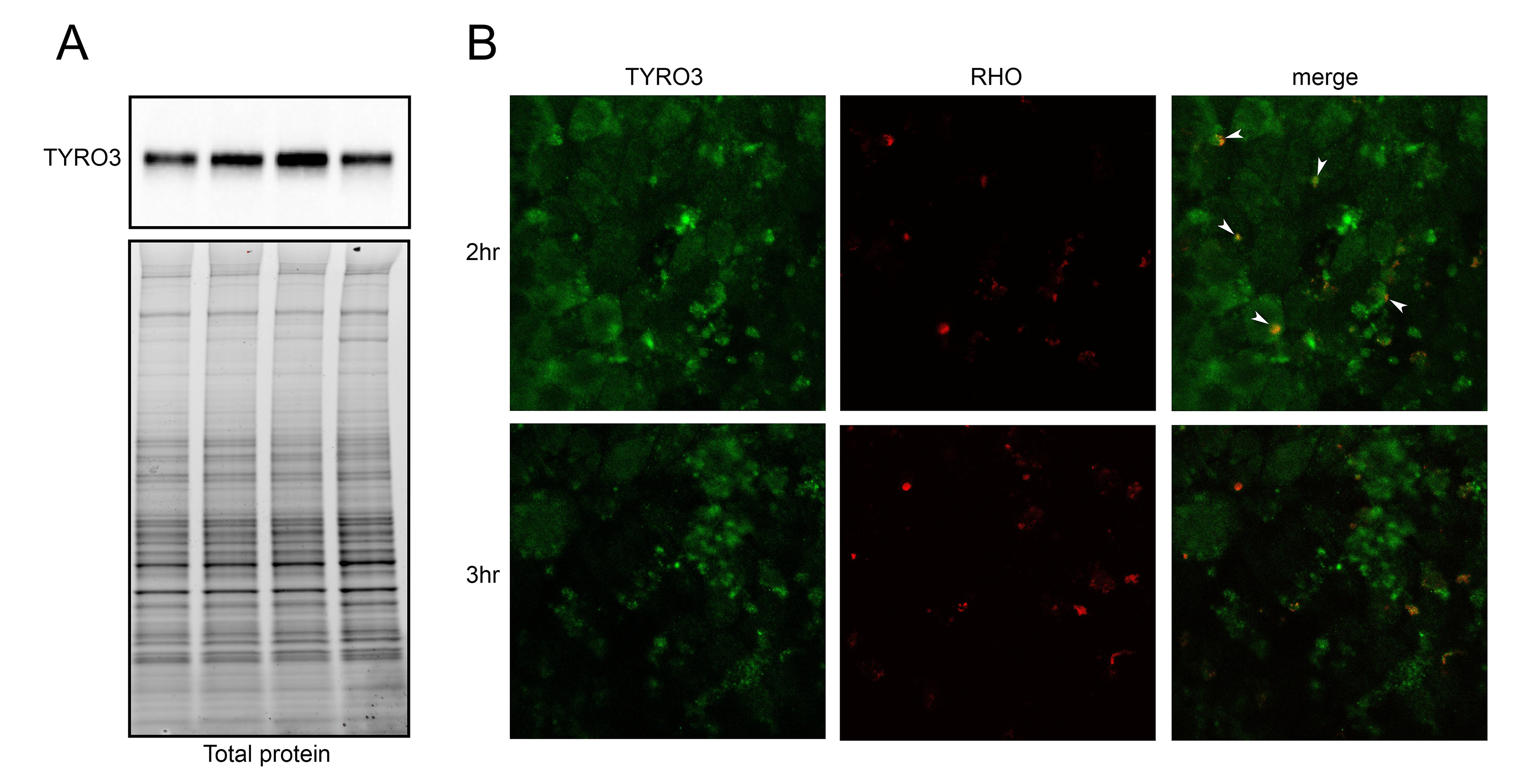

Supplement: S3 Fig — (A) Immunoblot for TYRO3 in four differentiated human primary RPE cell lines. The stain-free gel and chemiluminescent blot images were captured using a ChemiDoc MP Imaging system (Bio-Rad). (B) Confocal images of primary human RPE cells from the same experiment as in Fig 7A show persistent, but diminished, co-localization of endogenous TYRO3 and bovine POS at later time points. White arrowheads mark sites of co-localization of TYRO3 and POS. (TIF) [file pgen.1005723.s003.tif]

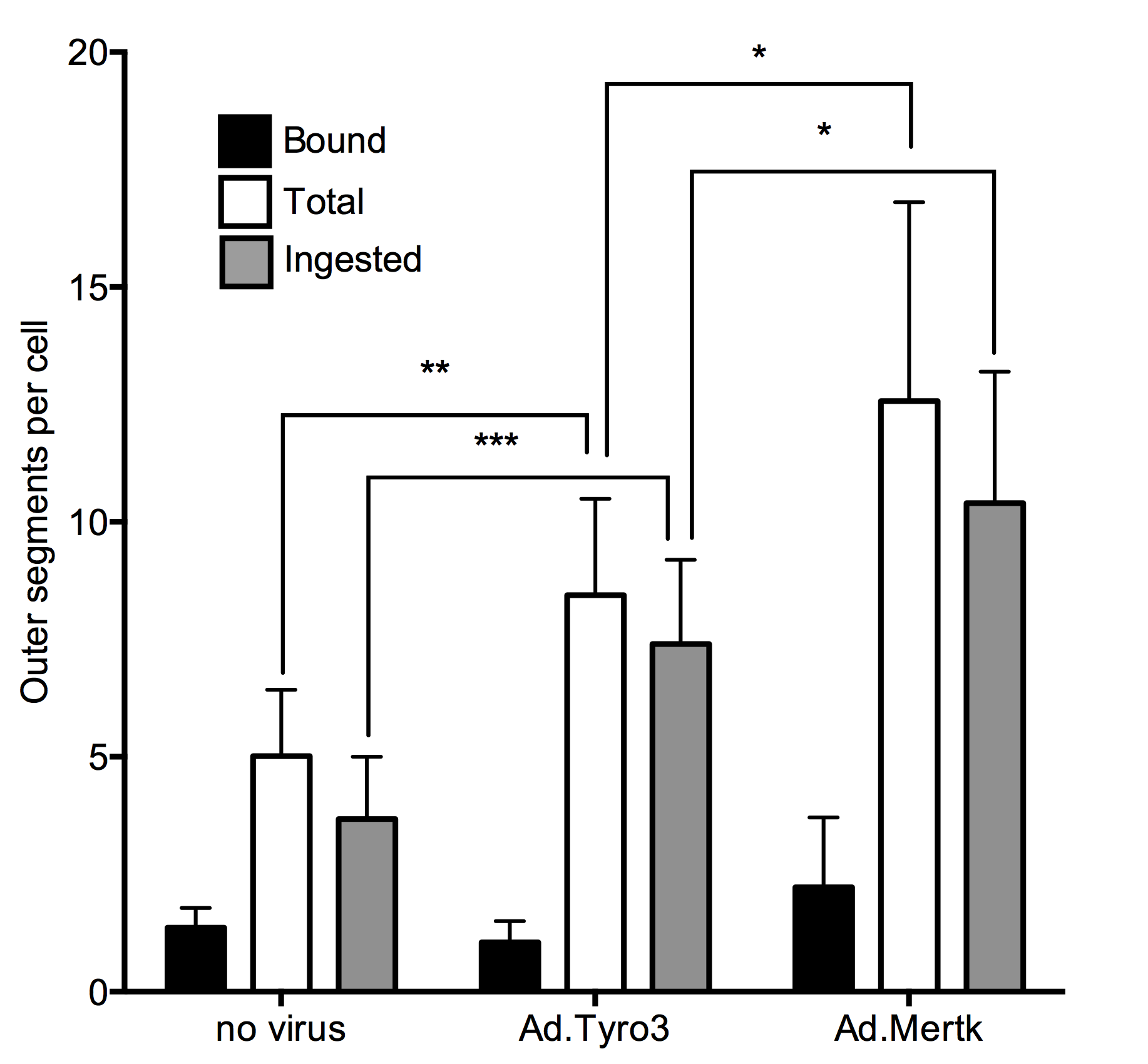

Supplement: S4 Fig — Primary mouse RPE cells were cultured, transduced with an adenoviral vector encoding murine Tyro3 or rat Mertk, and then assayed for their ability to phagocytize bovine outer segments (OS). Means ± SD are depicted. Tyro3 expression stimulates OS ingestion (total minus bound) compared to a no-virus control, as does rat Mertk. *** P ≤ 0.001, ** P ≤ 0.01, * P ≤ 0.05, calculated by two-way ANOVA with Bonferroni’s correction for multiple comparisons. (TIFF) [file pgen.1005723.s004.tiff]
