# Supplementary material for: Tyro3 Modulates Mertk-Associated Retinal Degeneration
Source: PLoS Genet. 2015 Dec 11;11(12):e1005723. doi: 10.1371/journal.pgen.1005723 (PMC4687644; doi:10.1371/journal.pgen.1005723)
Supplement: S1 Table — (PDF) [file pgen.1005723.s005.pdf]

S1 Table

Mouse gene	Human gene	
Phgr1	PHGR1	
Disp2	DISP2	
Knstrn	KNSTRN	
Ivd	IVD	
BHS1	BAHD1	
Chst14	CHST14	
Ccdc32	C15orf57	
Rpusd2	RPUSD2	
Casc5	CASC5	
Rad51	RAD51	
Rmdn3	RMDN3	
Gchfr	GCHFR	
DnaJc17	DNAJC17	
Gm14137	C15orf62	
Zfyve19	ZFYVE19	
Ppp1r14d	PPP1R14D	
Spint1	SPINT1	
Rhov	RHOV	
Vps18	VPS18	
Dll4	DLL4	
Chac1	CHAC1	
Ino80	INO80	
Exd1	EXD1	
Chp1	CHP	
Oip5	OIP5	
Nusap1	NUSAP1	
Ndufaf1	NDUFAF1	
Rtf1	RTF1	
Itpka	ITPKA	
Ltk	LTK	
Rpap1	RPAP1	
**Tyro3**	**TYRO3**	
Mga	MGA	
Mapkbp1	MAPKBP1	
Pla2g4b	PLA2G4B	
Jmjd7	JMJD7	
Ehd4	EHD4	
Pla2g4e	PLA2G4E	
Pla2g4d	PLA2G4D	
Pla2g4f	PLA2G4F	
Vps39	VPS39	
Tmem87a	TMEM87A	
Ganc	GANC	
Capn3	CAPN3	
Zfp106	ZFP106	
Snap23	SNAP23	
Lrrc57	LRRC57	
Haus2	HAUS2	
Stard9	STARD9	
Cdan1	CDAN1	
AK087617		inferred unnamed protein
mKIAA1300		hypothetical serine rich
AB001425		motor domain of KIF16A, partial cds
