# Supplementary material for: Tyro3 Modulates Mertk-Associated Retinal Degeneration
Source: PLoS Genet. 2015 Dec 11;11(12):e1005723. doi: 10.1371/journal.pgen.1005723 (PMC4687644; doi:10.1371/journal.pgen.1005723)
Supplement: S2 Table — (PDF) [file pgen.1005723.s006.pdf]

S2 Table

Chr	Position	Gene	dbSNP	Ref (B6)	MOLF_EiJ	PWK_PhJ	129P2_OlaHsd	A_J	AKR_J	BALB_cJ	C3H_HeJ	CAST_EiJ	CBA_J	DBA_2J	FVB_NJ	LP_J	NOD_ShiLtJ	WSB_EiJ
2	119797933	ENSMUSG0000006516	rs27424664	T	-	-	-	-	-	-	G	-	G	-	-	-	G	-
2	119798088	ENSMUSG0000006516	rs29499583	A	-	-	G	G	G	G	G	G	G	G	G	G	G	G
2	119798592	Tyro3	rs8240210	G	-	-	A	A	A	A	A	A	A	A	A	A	A	A
2	119799029	Tyro3	rs8255253	T	-	-	-	-	-	-	C	-	C	-	-	-	C	-
2	119799683	Tyro3	rs263371901	G	-	C	-	C	C	C	C	C	C	C	C	C	C	C
2	119799747	Tyro3	rs224575732	T	-	A	A	A	A	A	A	A	A	A	A	A	A	A
2	119799826	Tyro3	rs243468327	C	-	-	-	-	T	-	-	-	-	-	T	-	-	-
2	119800140	Tyro3	rs255479804	T	-	-	C	C	C	C	C	C	C	C	C	C	C	C
2	119800743	Tyro3	rs244453893	A	-	-	-	-	-	-	C	-	C	-	-	-	C	-
2	119800850	Tyro3	rs218598098	T	-	-	C	C	C	C	C	C	C	C	C	C	C	C
2	119800929	Tyro3	rs239028889	G	-	-	-	T	-	T	-	-	-	T	-	-	-	-
2	119801101	Tyro3	rs48422705	T	-	-	C	C	C	C	C	C	C	C	C	C	C	C
2	119801116	Tyro3	rs51559242	G	-	-	-	C	-	C	-	-	-	C	-	-	-	-
2	119801315	Tyro3	rs213239537	C	-	-	-	-	G	-	-	-	-	-	G	-	-	G
2	119801466	Tyro3	rs33556317	T	-	-	C	C	C	C	-	C	-	C	C	C	-	C
2	119801744	Tyro3	rs246979646	G	-	-	-	-	A	-	-	-	-	-	A	-	-	-
2	119801844	Tyro3	rs47419458	A	-	-	G	G	G	G	-	G	-	G	G	G	-	G
2	119801953	Tyro3	rs46154809	T	-	-	C	C	C	C	C	C	C	C	C	C	C	C
2	119802160	Tyro3	rs235095541	C	-	-	-	-	T	-	-	-	-	-	T	-	-	T
2	119802294	Tyro3	rs33488477	A	-	-	G	G	G	G	G	G	G	G	G	G	G	G
2	119802351	Tyro3	rs33131330	T	-	C	C	C	C	C	C	C	C	C	C	C	C	C
2	119802400	Tyro3	rs33216817	A	-	-	G	G	G	G	G	G	G	G	G	G	G	G
2	119802475	Tyro3	rs29672504	T	-	-	C	C	C	C	C	C	C	C	C	C	C	C
2	119802476	Tyro3	rs238933564	G	-	-	-	-	-	-	A	-	A	-	-	-	A	-
2	119802713	Tyro3	rs33331807	C	-	-	T	T	-	T	-	T	-	T	-	T	-	-
2	119803018	Tyro3	rs52186209	T	-	-	C	C	C	C	C	C	C	C	C	C	C	C
2	119803122	Tyro3	rs50911603	C	-	-	T	T	-	T	-	T	-	T	-	T	-	-
2	119803204	Tyro3	rs224269071	C	-	-	-	-	-	-	A	-	A	-	-	-	A	-
2	119803510	Tyro3	rs29968154	C	-	-	G	G	G	G	G	G	G	G	G	G	G	G
2	119803535	Tyro3	rs29671072	G	-	-	T	T	-	T	T	T	T	T	-	T	T	T
2	119803601	Tyro3	rs248638823	C	-	-	-	-	-	-	T	-	T	-	-	-	T	-
2	119803699	Tyro3	rs50066337	A	-	-	G	G	G	G	G	G	G	G	G	G	G	G
2	119803810	Tyro3	rs48813460	G	-	-	A	A	A	A	A	A	A	A	A	A	A	A
2	119803894	Tyro3	rs224904694	G	-	-	-	-	-	-	A	-	A	-	-	-	A	-
2	119804107	Tyro3	rs247663885	A	-	-	-	-	-	-	C	-	C	-	-	-	C	-
2	119804194	Tyro3	rs33503297	T	-	C	C	C	C	C	C	-	C	C	C	C	C	C
2	119804258	Tyro3	rs33556265	T	C	C	C	C	C	C	C	C	C	C	C	C	C	C
2	119804625	Tyro3	rs51099387	T	-	-	C	C	C	C	C	C	C	C	C	C	C	C
2	119804644	Tyro3	rs47293211	A	-	-	T	T	T	T	T	T	T	T	T	T	T	T
2	119804675	Tyro3	rs47273885	T	-	-	C	C	C	C	C	C	C	C	C	C	C	C
2	119804863	Tyro3	rs33639350	A	-	-	-	G	G	G	G	-	G	G	G	-	G	G
2	119804885	Tyro3	rs48181050	A	-	-	C	C	-	C	-	C	-	C	-	C	-	C
2	119804980	Tyro3	rs51148379	G	-	C	C	C	C	C	C	C	C	C	C	C	C	C
2	119805117	Tyro3	rs235319245	T	G	-	-	-	-	-	-	-	-	-	-	-	-	-
2	119805408	Tyro3	rs264164117	C	-	-	T	T	T	T	T	T	T	T	T	T	T	T
2	119805440	Tyro3	rs51838455	C	-	-	T	T	-	T	T	T	T	T	-	T	T	-
2	119805496	Tyro3	rs49553168	C	-	-	T	T	T	T	T	T	T	T	T	T	T	T
2	119805499	Tyro3	rs50686678	C	-	-	T	T	T	T	T	T	T	T	T	T	T	T
2	119805768	Tyro3	rs3725596	G	-	-	A	A	A	A	A	A	A	A	A	A	A	A
2	119805825	Tyro3	rs46502680	G	-	-	A	-	-	-	-	A	-	-	-	A	-	-
2	119805949	Tyro3	rs3726776	C	-	-	T	-	-	-	T	T	T	-	-	T	T	-
2	119805968	Tyro3	rs3726797	C	-	-	G	G	G	G	G	G	G	G	G	G	G	G
2	119806054	Tyro3	rs3653622	T	-	-	C	C	C	C	C	C	C	C	C	C	C	C
2	119806058	Tyro3	rs3653625	G	-	-	A	A	A	A	A	A	A	A	A	A	A	A
2	119806075	Tyro3	rs3654072	T	-	-	A	A	A	A	A	A	A	A	A	A	A	A
2	119806084	Tyro3	rs3654089	A	-	-	G	G	G	G	G	G	G	G	G	G	G	G
2	119806098	Tyro3	rs3654115	T	-	-	C	C	C	C	C	C	C	C	C	C	C	C
2	119806114	Tyro3	rs3654164	A	-	-	C	C	C	C	C	C	C	C	C	C	C	C
2	119806156	Tyro3	rs52347628	G	-	-	T	T	T	T	T	T	T	T	T	T	T	T
2	119806223	Tyro3	rs52461944	C	-	-	-	-	T	-	T	-	T	-	T	-	T	T
2	119806365	Tyro3	rs49266913	C	-	-	T	T	T	T	T	T	T	T	T	T	T	T
2	119806500	Tyro3	rs49350885	C	-	-	-	-	T	-	T	-	T	-	T	-	T	T
2	119806520	Tyro3	rs29819967	C	-	-	T	-	-	-	-	T	-	-	-	T	-	-
2	119806526	Tyro3	rs33057037	A	-	-	G	G	G	G	G	G	G	G	G	G	G	G
2	119806567	Tyro3	rs33275787	G	-	-	T	T	-	T	-	T	-	T	-	T	-	-
2	119806611	Tyro3	rs29587267	C	-	-	T	T	T	T	T	T	T	T	T	T	T	T
2	119806740	Tyro3	rs29517103	C	-	-	T	T	T	T	T	T	T	T	T	T	T	T
2	119806839	Tyro3	rs33340597	G	-	-	A	A	A	A	A	A	A	A	A	A	A	A
2	119807050	Tyro3	rs216678221	A	-	-	-	-	T	-	T	-	T	-	T	-	T	T
2	119807392	Tyro3	rs237104230	C	-	-	-	-	A	-	-	-	-	-	A	-	-	A
2	119807423	Tyro3	rs387437281	C	-	-	-	-	T	-	-	-	-	-	T	-	-	-
2	119807448	Tyro3	rs47734538	C	-	-	T	T	T	T	T	T	T	T	T	T	T	T
2	119808073	Tyro3	rs215110521	G	-	-	-	-	A	-	A	-	A	-	A	-	A	A
2	119808091	Tyro3	rs48974765	A	-	-	G	G	G	G	G	G	G	G	G	G	G	G
2	119808096	Tyro3	rs46259691	T	-	-	C	C	C	C	C	C	C	C	C	C	C	C
2	119808103	Tyro3	rs46513983	A	-	-	G	G	G	G	G	G	G	G	G	G	G	G
2	119808189	Tyro3	rs50893381	G	-	-	A	A	A	A	A	A	A	A	A	A	A	A
2	119808394	Tyro3	rs49289947	T	-	-	C	C	C	C	C	C	C	C	C	C	C	C
2	119808495	Tyro3	rs47972163	G	-	-	A	A	A	A	A	A	A	A	A	A	A	A
2	119808504	Tyro3	rs49338907	A	-	-	C	C	C	C	C	C	C	C	C	C	C	C
2	119808558	Tyro3	rs45950089	T	-	-	C	C	C	C	C	C	C	C	C	C	C	C
2	119808664	Tyro3	rs46515695	T	-	-	C	-	C	-	C	C	C	-	C	C	C	C
2	119808669	Tyro3	rs51574702	T	-	-	A	-	A	-	A	A	A	-	A	A	A	A
2	119808734	Tyro3	rs224708901	G	-	-	-	-	A	-	A	-	A	-	A	-	A	A
2	119808815	Tyro3	rs51159114	G	-	-	-	A	-	A	-	-	-	A	-	-	-	-
2	119808884	Tyro3	rs48916544	A	-	-	-	G	G	G	G	-	G	G	G	-	G	G
2	119808898	Tyro3	rs46349139	T	-	-	C	C	C	C	C	C	C	C	C	C	C	C
2	119808971	Tyro3	rs50827695	A	-	-	G	G	G	G	G	G	G	G	G	G	G	G
2	119808972	Tyro3	rs50718304	A	-	-	G	G	G	G	G	G	G	G	G	G	G	G
2	119809170	Tyro3	rs48752764	C	-	-	A	A	-	A	-	A	-	A	-	A	-	-
2	119809268	Tyro3	rs50533867	T	-	-	A	A	A	A	A	A	A	A	A	A	A	A
2	119809269	Tyro3	rs46026954	G	-	-	T	T	T	T	T	T	T	T	T	T	T	T

2	119809346	Tyro3	rs47104675	A	-	-	G	G	G	G	G	G	G	G	G	G	G	G
2	119809348	Tyro3	rs259818895	C	-	-	T	-	-	-	-	T	-	-	-	T	-	-
2	119809583	Tyro3	rs49435215	C	-	-	T	T	T	T	T	T	T	T	T	T	T	T
2	119809620	Tyro3	rs49869472	A	-	-	T	T	-	T	T	-	T	T	-	T	T	~
2	119809755	Tyro3	rs265189593	C	-	-	T	T	T	T	T	T	T	T	T	T	T	T
2	119809756	Tyro3	rs229413765	A	-	-	G	G	G	G	G	G	G	G	G	G	G	G
2	119809757	Tyro3	rs249059828	T	-	C	C	C	C	C	C	C	C	C	C	C	C	C
2	119809820	Tyro3	rs50990498	C	-	-	T	T	T	T	T	T	T	T	T	T	T	T
2	119809832	Tyro3	rs46155772	G	-	-	-	A	-	A	-	-	-	A	-	-	-	-
2	119809898	Tyro3	rs48728041	T	-	-	C	C	C	C	C	C	C	C	C	C	C	C
2	119810451	Tyro3	rs4136004	C	-	-	T	-	T	-	T	T	T	-	T	T	T	T
2	119810913	Tyro3	rs27424661	C	-	-	-	-	T	-	-	-	-	-	T	-	-	T
2	119810952	Tyro3	rs27424660	C	-	-	-	T	-	T	-	-	-	T	-	-	-	-
2	119811012	Tyro3	rs27424659	G	-	-	-	-	A	-	-	-	-	-	A	-	-	A
2	119811112	Tyro3	rs46218373	G	-	-	-	A	-	A	-	-	-	A	-	-	-	-
2	119811187	Tyro3	rs27424658	G	-	-	-	-	A	-	A	-	A	-	A	-	A	A
2	119811196	Tyro3	rs27424657	G	-	-	A	A	A	A	A	A	A	A	A	A	A	A
2	119811220	Tyro3	rs27424656	C	-	-	A	A	A	A	A	A	A	A	A	A	A	A
2	119811346	Tyro3	rs45988723	C	-	-	T	T	T	T	T	T	T	T	T	T	T	T
2	119811356	Tyro3	rs46825732	C	-	-	T	T	T	T	T	T	T	T	T	T	T	T
2	119811366	Tyro3	rs47585089	G	-	-	C	C	C	C	C	C	C	C	C	C	C	C
2	119811410	Tyro3	rs46481213	C	-	-	T	T	T	T	T	T	T	T	T	T	T	T
2	119811552	Tyro3	rs45849779	G	-	-	C	C	C	C	C	C	C	C	C	C	C	C
2	119811883	Tyro3	rs27424655	A	-	-	T	-	T	-	T	T	T	-	T	T	T	T
2	119811973	Tyro3	rs27424654	G	-	-	-	A	-	A	-	-	-	A	-	-	-	-
2	119812254	Tyro3	rs27424653	C	-	-	T	T	-	T	-	T	-	T	-	T	-	-
2	119812397	Tyro3	rs27424651	G	-	-	A	A	A	A	A	A	A	A	A	A	A	A
2	119812410	Tyro3	rs27424650	A	-	-	-	C	-	C	-	-	-	C	-	-	-	-
2	119812556	Tyro3	rs13459234	C	-	-	G	G	G	G	G	G	G	G	G	G	G	G
2	119812697	Tyro3	rs27424649	C	-	-	T	-	-	-	-	T	-	-	-	T	-	-
2	119813146	Tyro3	rs251734907	C	-	-	-	-	T	-	T	-	T	-	T	-	T	T
2	119813234	Tyro3	rs27424648	C	-	-	T	T	T	T	T	T	T	T	T	T	T	T
2	119813443	Tyro3	rs33222875	C	-	-	T	-	-	-	-	T	-	-	-	T	-	-
2	119813846	Tyro3	rs246659726	C	-	-	-	-	T	-	T	-	T	-	T	-	T	T
2	119815246	Tyro3	rs27424647	A	-	-	G	G	G	G	G	G	G	G	G	G	G	G
2	119815560	Tyro3	rs240396163	G	-	-	-	-	C	-	C	-	C	-	C	-	C	C
2	119815715	Tyro3	rs253025997	C	-	-	-	-	T	-	T	-	T	-	T	-	T	T
2	119815935	Tyro3	rs33115787	C	-	-	A	-	-	-	-	A	-	-	-	A	-	-
2	119816063	Tyro3	rs265168874	T	-	-	-	-	C	-	C	-	C	-	C	-	C	C
2	119816386	Tyro3	rs51179699	C	-	-	T	T	-	T	-	T	-	T	-	T	-	-
2	119816488	Tyro3	rs33489247	A	-	-	G	-	-	-	-	-	-	-	-	G	-	-
2	119816626	Tyro3	rs27424646	C	-	-	-	-	T	-	T	-	T	-	T	-	T	T
2	119816822	Tyro3	rs47863852	G	-	T	C	C	C	C	C	C	C	C	C	C	C	C
2	119816824	Tyro3	rs48021866	C	-	G	G	G	G	G	G	G	G	G	G	G	G	G
2	119816861	Tyro3	rs13459232	G	-	-	-	-	A	-	A	-	A	-	A	-	A	A
2	119817077	Tyro3	rs27424645	G	-	-	A	-	-	-	-	-	-	-	-	A	-	-
2	119817081	Tyro3	rs27424644	T	-	-	-	C	-	C	-	-	-	C	-	-	-	-
2	119817664	Tyro3	rs33708304	G	-	-	-	C	-	C	-	-	-	C	-	-	-	-
2	119817684	Tyro3	rs27424643	G	-	-	A	-	-	-	-	-	-	-	-	A	-	-
2	119817696	Tyro3	rs13459231	C	-	-	-	-	T	-	T	-	T	-	T	-	T	T
2	119817751	Tyro3	rs13459233	T	-	-	C	C	C	C	C	C	C	C	C	C	C	C
2	119818007	Tyro3	rs27424642	T	-	-	C	C	C	C	C	C	C	C	C	C	C	C

data were downloaded from http://www.sanger.ac.uk/resources/mouse/genomes/

'-' indicates identity with C57BL/6 reference

MOLF is identical to B6 at 142 of 144 positions; PWK is identical at 135 of 144 positions; 129P2 is identical at 44 of 144 positions.

Highlighted are 89 positions at which the B6 reference and MOLF (and sometimes PWK) share an allele that is different from the allele present in most or all other strains in the table.
