# Supplementary material for: Tyro3 Modulates Mertk-Associated Retinal Degeneration
Source: PLoS Genet. 2015 Dec 11;11(12):e1005723. doi: 10.1371/journal.pgen.1005723 (PMC4687644; doi:10.1371/journal.pgen.1005723)
Supplement: S3 Table — (PDF) [file pgen.1005723.s007.pdf]

S3 Table

STR	Forward Primer	Reverse Primer
D2Mit22	GCTCCCTTTCCTCTTGAACC	GGGCCCTTATTCTATCTCCC
D2Mit168	CTCACAGACACTGCACTATTACACA	TGTTCCTGCTATTGTTTTGGG
D2Mit194	TGGAATTCCAAAGTCAAGGG	GGGAAGAATGGGGGAAGTTA
D2Mit255	GCAAGTGTGATCTGGGTGC	TGAGCACACTTACACTGTGGTG
D2Mit164	TCTCTGCTAATTAAGTTGAAGAGTGC	ACCAGTGTGTGTTTGTATGATGTG
D2Mit397	TGATGAAGGTTCTTTTTCTCCC	CCACAGTTGGTAATTATCTGGC
D2Mit445	CCTATACACGCACACACAGACA	ATGCCCTGCTTGCTATTGTT
D2Mit395	AGGTCAGCCTGGACTATATGG	AGCATCCATGGGATAATGGT
D2Mit62	GGATACCGTTTGGAAAGTAAACC	GCAAGAAGCACAGGAGGC
D2Mit101	ATAATTCCTGATTTGCTGTTTGTG	ACATGAAGCCTAGAGGGTGC
D2Mit206	TGTCAGAACTGGACAATGTCG	ATGATAACAGACACTAATGATTAGGGC
D2Mit94	GGCTTCGACCCTGGTTTTAG	TGAAAGTTCAGATGACCACAGG
**SNP**		
rs27439123	ACTGGTGAGGTTAGCTGTGC	TCTTCCCTCTGTTGCCTAAG
rs27454014	GGGGTACTGCACTGAACAAG	ACCTCTTGCATGGCTTCTC
rs3669873	TATACTTCCACCCCAAAAGG	TCAGCCCTTCAGCTCTTTTA
rs27443946	CCCAGCAACTCACTTAGCAT	CTTGCTCGGCTACCTTTTT
rs3686523	GGAGTCTATGGGGGAAGAGA	GCATGCATCTTAGTCCCTGA
rs8240210	CTGCTGCTCAGGAGAATCC	GCACAGTAGGCACTCCATAAA
rs27424653†	GGAGGCACTGTTGCAGATAC	AAATTTCTTCCCGCTAAAGG
rs3684717	TTGGCTTTGGTCCTTCTG	TGCATGCAAAATGGTTTTAG
rs27440128	TTCTTCTCTTCCTGCCCTGT	ACCTCCCGTACTTGTTTCCT
rs3144592	AAGCTGGATCCTTGCTGTG	AGAGCCCAGCTCAGTGTTAG
rs3144638	CAGATGAGCACTGTTTGTGTG	CTACAGCTGGGAAATTCAGG
rs3702275	CAGAGAAGCTGCCAAAAGAG	GAGGACAATGTTCAGGGATG
rs27427063	GCCTGCTGATCTCTTTTGTG	TTTGGTGTGCTCAGTGTTTG
rs27504920	CCCCCAGTAATTGTGCTAAG	TGGTCTATTCAGGCAATTATGAC
***mTyro3* ƒ**		
**sequencing**		
5002; 4816	ATGGCGCTGAGGCGGAGCAT	ACAGCTGAAAAGGGGCATT
4817; 4156	GAAGATGACCGTGTCTCAGG	TGCTTGAAGGCGAACAATGG
5026; 4818	GTCGAAGGTGTGCCATTCTT	GAAATTCTGAGGAGCTCTGG
4819; 4820	AACGATCTCCAGCTACAACG	GCTTTTCCTTCAATTCATCG
5028; 5035	CATCCTGCTTCGGAAGAGAC	CTTCACTGCCACTTTCACGA
5046; 5045	CGATGAATTGAAGGAAAAGC	CCAAAATCAGCCACACACAC
4823; 4824	TTGCATGAAGGAGTTTGACC	AGATGTACCGAGAGTCACTGG
5029; 5042	CCATATGCTGGCATTGAAAA	GGGCTGAAGATGTACCGAGA
4825; 4826	TGCCGAGATTTACAACTACC	TGCTACCCTCCCTTACTGC
**qRT-PCR ƒ**		
4493; 4494	GTGAAGCCCGCAACATAA	CAGTACAGGAATGCAGCAGA
4670; 4671 (*Eef1a1*)	GCAGAAGAAAAGGCAATGGT	AGGCCGTAGTACAGCAGGAT

ƒVollrath lab identifiers
† near *mTyro3* exon 15
