# Supplementary material for: Tyro3 Modulates Mertk-Associated Retinal Degeneration
Source: PLoS Genet. 2015 Dec 11;11(12):e1005723. doi: 10.1371/journal.pgen.1005723 (PMC4687644; doi:10.1371/journal.pgen.1005723)
Supplement: S4 Table — (PDF) [file pgen.1005723.s008.pdf]

S4 Table

SNP/STR	chr2 coordinates*	Notes†
D2Mit22	151799041	rs262340220
D2Mit168	144822124	rs264258584
D2Mit194	143931545	rs227522187
Mertk	128698997-128802188	full transcript
D2Mit255	123391787	rs227455427
D2Mit164	123390079	rs260314159
D2Mit397	122826629	rs257766288
rs27439123	122024278	**C**/T
rs27454014	121655895	**A**/T
D2Mit445	121297614	rs226388668
rs3669873	120791045	**C**/T
rs27443946	120417283	**T**/G
rs3686523	120044013	**G**/T
rs8240210	119798592	**G**/A
rs27424653	119812254	**C**/T
Tyro3	119797740-119818103	full transcript
D2Mit395	119524934	rs253737247
rs3684717	119069944	**A**/G
rs27440128	118861643	**G**/A
rs3144592	118691878	**T**/G
rs3144638	118559508	**A**/C
D2Mit62	118112472	rs252700598
rs3702275	116947245	**T**/C
rs27427063	115818013	**C**/T
D2Mit101	115079497	rs246380276
rs27504920	112791086	**G**/A
D2Mit206	106909940	rs236046102
D2Mit94	80175611	rs244364794

* from GRCm38/mm10 Assembly

† Alleles for SNPs are shown with reference allele bolded. For STRs the rs# is shown.
