# Supplementary material for: Tyro3 Modulates Mertk-Associated Retinal Degeneration
Source: PLoS Genet. 2015 Dec 11;11(12):e1005723. doi: 10.1371/journal.pgen.1005723 (PMC4687644; doi:10.1371/journal.pgen.1005723)
Supplement: S5 Table — (PDF) [file pgen.1005723.s009.pdf]

S5 Table

Antibody against	Source	Application (origin of antigen)	Dilution
TYRO3	Cell Signaling	IF (mouse)<br>IB (mouse)	1:50<br>1:1000
TYRO3	abcam (ab109231)	IF (human)<br>IB (human)	1:200<br>1:1000
RLBP1	John Saari	IB (mouse)	1:40,000
cytokeratin	abcam	IF (mouse)	1:100
γ-tubulin	Sigma-Aldrich	IB (mouse)	1:5000
rhodopsin (rho 4D2)	Robert Molday	IF (bovine)	1:200
